# Automated real-time feeding control for microbial electrolysis cell-anaerobic digestion systems using finite state machine

**DOI:** 10.1038/s41598-026-57116-x

**Published:** 2026-06-20

**Authors:** Harvey Rutland, Kyle Bowman, Thomas Fudge, Emma Crossley, Godfrey Kyazze, Haixia Liu, Jiseon You

**Affiliations:** 1 School of Computer Science, Electrical and Electronic Engineering, and Engineering Maths, University of Bristol, BS8 1UB Bristol, UK; 2 WASE, Bristol, UK; 3 School of Life Sciences, University of Westminster, 115 New Cavendish Street, W1W 6UW London, UK; 4 School of Computing and Creative Technologies, University of the West of England, Coldharbour Ln, BS16 1QY Bristol, UK; 5 School of Engineering, University of the West of England, Coldharbour Ln, BS16 1QY Bristol, UK

**Keywords:** Microbial electrolysis cell, Anaerobic digestion, Biosensor led control, Finite state machine, Biological techniques, Biotechnology, Energy science and technology, Engineering

## Abstract

This study investigates the use of biosensor-led control in Microbial Electrolysis Cell-Anaerobic Digestion (MEC-AD) systems to enhance operational stability. Traditional methods depend on human operators to interpret data and adjust processes, whereas this research employed a current threshold-based Finite State Machine (FSM) for automated control in lab-scale, single-chamber MEC-AD reactors operated continuously for four months. By monitoring current draw as an indirect electrochemical proxy for microbial substrate-utilisation activity, the study facilitated real-time control of feeding events based on current responses to organic loading. Results show that, under the tested lab-scale conditions, this method enabled adjustment of feed volume and timing in response to changes in system conditions and microbial activity. Using an FSM provided a structured framework that links current responses to feeding events and defined system states, enabling predictable management of the MEC-AD process. Using molasses as feedstock, the research demonstrates effectiveness across reactors with varied hydraulic retention times at lab scale, indicating potential for further investigation into scalability and automation. This approach offers a promising alternative for optimising the performance of continuous operation AD systems, ensuring better control and lower risk of overload failures.

## Introduction

Improper treatment of biomass residue can lead to significant environmental issues, and proper disposal methods can be costly. Operating industrial anaerobic digestion (AD) facilities is expensive and presents challenges in remote locations. Consequently, methods for remote operation are becoming increasingly popular, aligning with the advancements of Industry 4.0, which emphasises the use of automation and real-time data monitoring to enhance industrial processes^1^. AD offers a promising solution by treating organic biomass and producing renewable energy. This process involves microorganisms that break down substrates anaerobically in four phases: hydrolysis (complex organics broken down into simple sugars, amino acids, and long-chain fatty acids), acidogenesis (producing H_2_, CO_2_, alcohols and volatile fatty acids (VFAs)), acetogenesis (VFAs and alcohols to acetate, H_2_ and CO_2_), and methanogenesis (producing CH_4_). AD technology efficiently converts waste into biogas, which mainly consists of CH_4_ (50–75%), CO_2_ (25–50%), and trace amounts of N_2_, H_2_S, and H_2_^2^. Microbial Electrolysis Cells (MECs), part of a technology group termed bioelectrochemical systems, efficiently catalyse electrochemical reactions using microorganisms^3^. Previous studies have reported that integrating MECs into AD systems can enhance organic treatment rates and methane production under certain operating conditions^4,5^. However, the extent of this contribution depends on reactor configuration, applied potential, microbial community structure, and operating conditions. Applying a small potential difference across the MEC system accelerates the breakdown of organic substrates, positioning MECs as a promising technology for small-scale, sustainable biogas production^6^.

Recent research on MECs indicates that the electrical current drawn by the MEC module is closely associated with bacterial activity within the system^7^. This relationship reflects the interaction between microorganisms and electrochemical processes. As bacteria in MECs metabolise organic substrates, they transfer electrons to the anode, which are subsequently donated to chemical and microbial species at the cathode, generating measurable current. Fluctuations in current draw are therefore consistent with changes in electrochemical activity and may indicate variations in microbial metabolism and electrode performance^8,9^.

However, current represents an indirect electrochemical signal rather than a direct measurement of microbial community structure or health. Under stable and uninhibited conditions, current trends can provide useful insight into substrate utilisation dynamics. In contrast, during inhibitory states or process imbalance, soluble intermediates may accumulate without a proportional increase in current, leading to potential decoupling between electrochemical response and overall digestion performance. Real-time monitoring of module current draw offers a non-invasive method to assess system behaviour, leveraging existing infrastructure without requiring additional sensor hardware. Current measurements have been used in various studies to evaluate electrochemical performance within MEC systems^10^. When interpreted alongside broader operational indicators, this monitoring capability can support timely adjustments and contribute to improved operational stability.

In this study, the term “biosensor-led control” refers to a feedback control strategy in which real-time MEC current is used as an indirect biosensor signal of electrochemical and substrate-utilisation activity to govern reactor feeding. Specifically, the measured current is interpreted by a finite state machine (FSM), which initiates feeding events when the signal falls below a defined threshold. Thus, “biosensor-led” here does not imply direct biological sensing of microbial composition, but rather process control guided by an electrochemical proxy derived from microbial activity.

To achieve feeding in AD systems, timer-based feeding control is commonly used, where the strength of the organic feed is monitored to determine suitable feed rates. This fixed-interval feeding has been investigated in multiple studies to understand how varying the dosing frequency impacts system performance, with the reactor’s health monitored through regular sampling^11,12^. Studies have found that lower-frequency dosing can make bacteria more resilient to organic overloading than communities developed under continuous feeding conditions. An alternative to timer-based or dosed feeding in AD systems is the use of continuous feeding methods. Continuously fed reactors are designed to maintain a consistent organic loading rate, avoiding the peaks and troughs associated with batch dosing and potentially leading to more uniform biogas production. However, system feeding rates need to be monitored and adjusted to ensure overloading is avoided^13^. Dosing and continuous feeding methods, however, are both fixed controls that do not leverage system feedback to adjust feeding rates; this may be important in applications where feed strength can vary. Other work has been conducted to explore the feasibility of optimisation through adaptive feeding methods in simulation, enabling variations in time intervals and dosing levels to tailor biogas production. A broader range of instrumentation and control strategies has been investigated for anaerobic digestion systems, including model-based, adaptive, and sensor-driven approaches^14^. Non-linear modeling techniques were used to determine how changes in substrate strength could influence biogas yield, facilitating adjustments to drive non-fixed interval conditions^15^. An overview of selected control methods relevant to the present study is provided in Table 1.

**Table 1:** Comparative analysis of control strategies for AD feeding control, highlighting trade-offs motivating the FSM approach.

**Control method**	**Adaptability**	**Real-world feasibility**	**Complexity**	**Responsiveness**
PID Control	**Feedback-based model**; limited for non-linear systems, requiring frequent retuning and prone to windup.	Challenging in AD due to time delays and non-linearity; poor tuning risks instability^16^.	Low computational cost but difficult to robustly implement in AD systems.	Effective for small deviations; slow response to abrupt changes^17^.
AI-Based Control	**Data-driven model**; adapts to complex, non-linear behaviour using learned relationships^18^.	Requires high-quality data, domain expertise, and safeguards for deployment^19^.	High complexity due to training, validation, and model maintenance^20^.	Fast for learned patterns but unpredictable under unseen disturbances.
FSM Control	**Rule-based model**; adapts via real-time state transitions using system feedback.	Practical and modular; easily updated without full redesign^21^.	Low complexity; relies on simple conditional logic.	Rapid response to defined states; minimises unnecessary intervention^22^.

In full-scale anaerobic digestion systems, process control is commonly based on periodic monitoring of parameters such as pH, alkalinity, volatile fatty acid (VFA) concentration, FOS/TAC ratio, biogas production rate, and methane composition^23^. Because many conventional digesters operate at relatively long hydraulic retention times (HRT), system dynamics are often comparatively slow, and manual or low-frequency corrective actions can be sufficient to maintain stable operation. However, these approaches may be less suitable for smaller-scale, intensified, or high-rate systems, where substrate availability, microbial activity, and electrochemical response can change over shorter timescales. In such systems, real-time feedback signals may provide additional value by enabling feeding decisions to respond directly to reactor behaviour rather than relying solely on fixed schedules or delayed offline measurements.

AD is a complex biological process influenced by fluctuations in microbial activity and organic loading, complicating optimal feeding control. While feedback control with a PID controller is well-established and comparatively straightforward to implement, it generally requires optimal set points, these control methods can be difficult to robustly implement in non-linear systems such as AD where responses to control changes also have an inherent time-delayed behaviour^24,25^. Consequently, PID controllers can be slow to react to sudden changes, risking overfeeding or underfeeding which can lead to permanent augmentation of system dynamics. In contrast, AI-based approaches, such as the use of machine learning, can adapt intelligently to varying digester conditions by identifying complex patterns in operational data. For instance, trained predictive models could be used to predict impacts of changing organic loads and adjust feeding proactively. However, these methods require datasets to train the models on, which reflect a diverse representation of the operating environment and the relationship of the different features under given operating conditions and microbial dynamics. Caution towards using black-box AI controllers is common, as reliability often outweighs potential adaptability and automation, as such implementation of such methods may look to use ML-based predictions in conjunction with other levels of contingency in continuous operation^26,27^. Consequently, despite the potential benefits of AI-driven control, most commercial biogas facilities implement more basic control methods over AI assisted automation. FSMs offer a conceptual model for designing operational logic based on system feedback^28^. A FSM consists of defined states representing different system conditions, with specific rules governing transitions based on inputs or feedback. Applying an FSM to MEC-AD systems can replace timer-driven feed control by initiating feeding events when bacterial activity decreases, as indicated by current draw. This allows feeding intervals to vary based on microbial conditions within the reactor, enabling precise control and system diagnostics.

This study presents a novel approach to operating MEC-AD systems by employing a FSM that uses real-time current draw as an electrochemical feedback signal to initiate feeding events using molasses feedstock (COD: 950 g/L). Experiments were conducted for a period exceeding 25 days across three reactor setups operated under varying feeding methods and substrate conditions, resulting in differing organic loading rates (OLR) under FSM control. By using bacterial electrochemical response as a feedback signal to determine dosing intervals, the approach enables adaptive ramp-up during early operational stages and reduces reliance on manual intervention. Continuous monitoring of module current draw supports controlled feeding while also providing diagnostic insight into system behaviour and potential disturbances.

The manuscript is structured as a staged validation of the FSM-based feeding strategy. An initial startup phase evaluates adaptive ramp-up behaviour under biosensor control. This is followed by a steady-state recovery phase assessing robustness to disturbance and threshold recalibration. Finally, controlled feed-strength variation experiments in two reactors examine controller performance under dynamic loading conditions, collectively demonstrating the operational stability and adaptability of the proposed control framework.

## Materials and methods

### Reactor and control hardware setup

The reactors were fabricated from polycarbonate and operated as lab-scale continuously stirred tank reactors with a working volume of 2 L (2.6 L total volume). Temperature was maintained at 35 $^\circ$C using a temperature-controlled heated cabinet. Feed was delivered via a peristaltic pump, and effluent was removed through a gravity-draining outlet incorporating a U-bend to maintain constant liquid level. The MEC modules were suspended within the reactor volume, and mixing was provided using magnetic stirrers to ensure homogeneous digestate conditions.

Electrode designs were modelled after those described in^29^, using carbon based material for the anode and stainless steel for the current collectors at the cathode, as reported in^30^. The MECs were operated in a two-electrode configuration using the logging power supply, with an applied cell voltage of 1 V across the anode–cathode pair, following electrode configurations reported in prior works^31,32^. A reference electrode was not incorporated in the reactor design, as the primary objective of this study was to evaluate biosensor-led process automation under conditions representative of scalable system architectures rather than to perform detailed electrochemical characterisation. Maintaining a fixed applied potential simplified hardware requirements and improved practical deployability for long-duration operation. The applied voltage was therefore held constant throughout experimentation to ensure comparability of current responses over time and to enable the use of current as a consistent control input within the FSM.

**Fig. 1:**
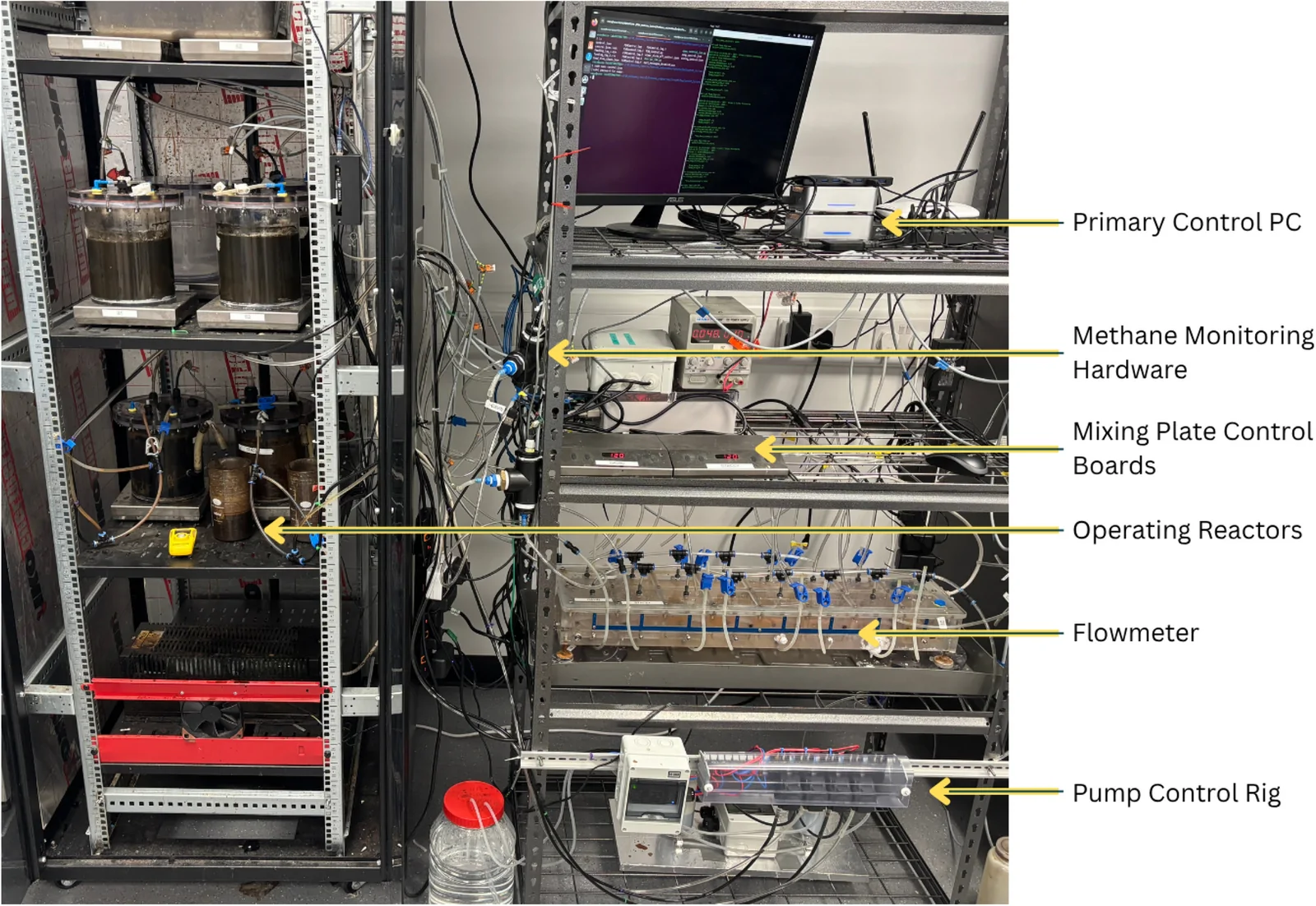
Annotated photograph of the experimental setup showing the complete MEC-AD automation rig. Key components are labelled: primary control PC running the FSM Python server, methane monitoring hardware, mixing plate control boards, operating reactors (2 L polycarbonate vessels), wet tip flowmeters for biogas measurement, and the pump control rig housing peristaltic pumps and microcontroller hardware.

**Fig. 2:**
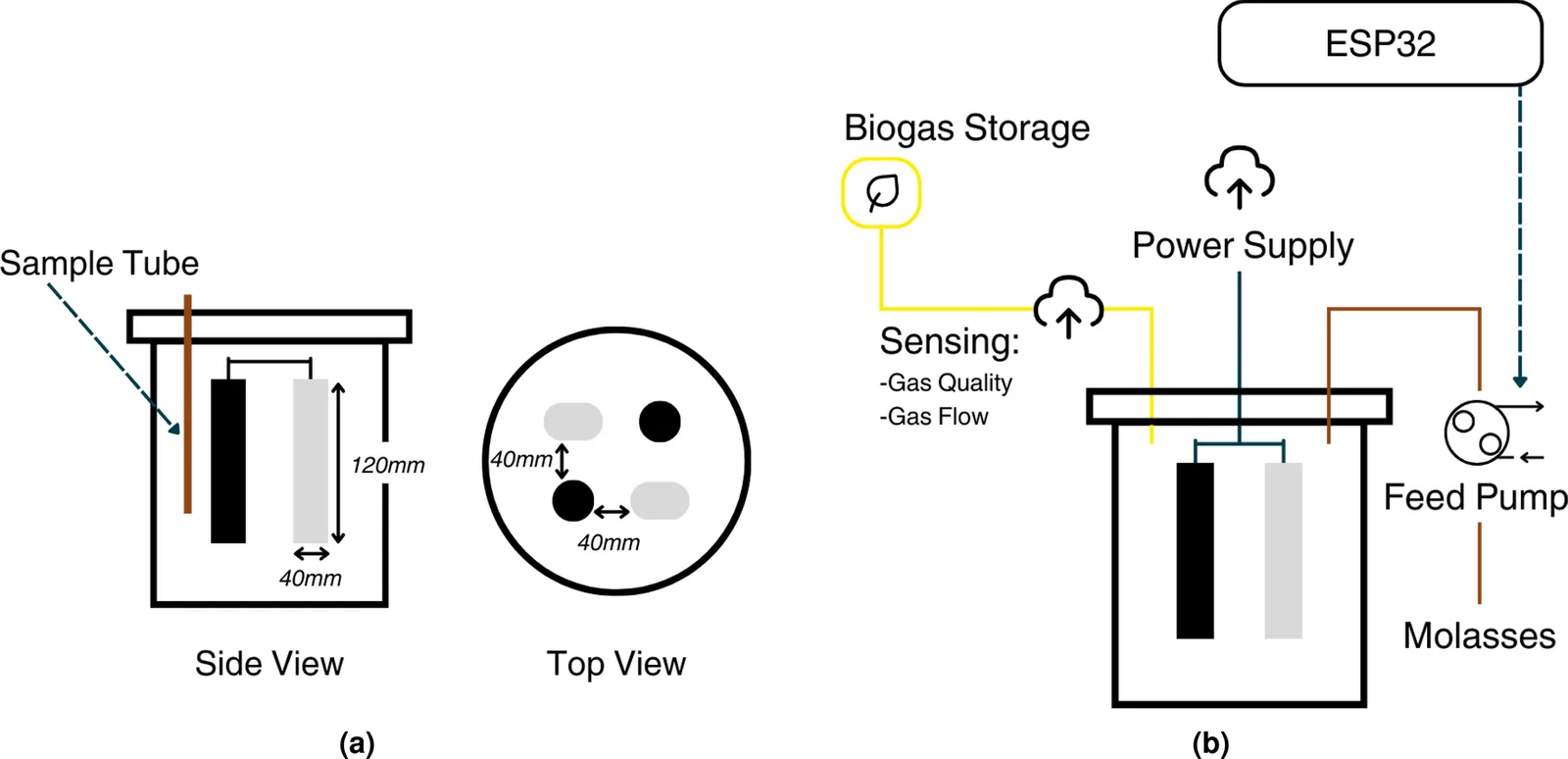
Physical configuration of the MEC-AD system: (**a**) Electrode geometry and spacing, comprising two anodes and two cathodes; (**b**) lab-scale reactor hardware layout.

Further details listing the dimensions of the module and electrode spacing are provided in Fig. 2(a). An outline of the reactor hardware setup is presented in Fig. 2(b), utilising a logging power supply (Rohde and Schwarz NGP814). A photograph of the complete experimental setup is provided in Fig. 1.

#### Electrode acclimation and biofilm establishment

Electroactive biofilms were established by immersing the anode and cathode assemblies in anaerobic digestate under mesophilic conditions (35 $^\circ$C). During acclimation, the MEC module was operated under applied polarisation using the same two-electrode configuration as used during reactor experiments, with an applied cell voltage of 1 V. Maintaining the electrodes under potential during this period was intended to stimulate the enrichment of electroactive microbial populations capable of extracellular electron transfer.

Although the system was energised throughout acclimation and qualitative current responses were observed following substrate additions, current was not continuously logged or quantitatively analysed during this phase. Acclimation was considered operationally successful when reproducible current transients were visually observed following substrate exposure (data not shown). Detailed electrochemical characterisation of the biofilm development phase was outside the scope of this study and represents an area for future investigation.

This pre-acclimation step was implemented to reduce variability during subsequent automated-control experiments by ensuring that anodic biofilms were established prior to initiating FSM-controlled feeding.

### Design of control architecture

The reactors were supplied with feed via a peristaltic pump and were controlled using an Adafruit MCP4728 Digital to Analogue Converter. Control of the Digital to Analogue Converter was managed by an Adafruit Feather ESP32. Top-level state control was executed through a Python server running on an Ubuntu PC.

Live current data was collected via two methods during experimentation. Initially, current was monitored using INA219 current sensors running on an Adafruit Feather board. After the initial phase of the experiment, the power supply and current monitoring were upgraded to use the Rohde and Schwarz NGP814 for improved reliability. Pump controls were transmitted to the ESP32 over a Flask server. This enabled pumps to be turned on for a set period when feeding was required. This setup allows for automated activation of pumps at a specified time, sent from the Python script to the microcontroller, which initiates the pump for that specified duration.

**Fig. 3:**
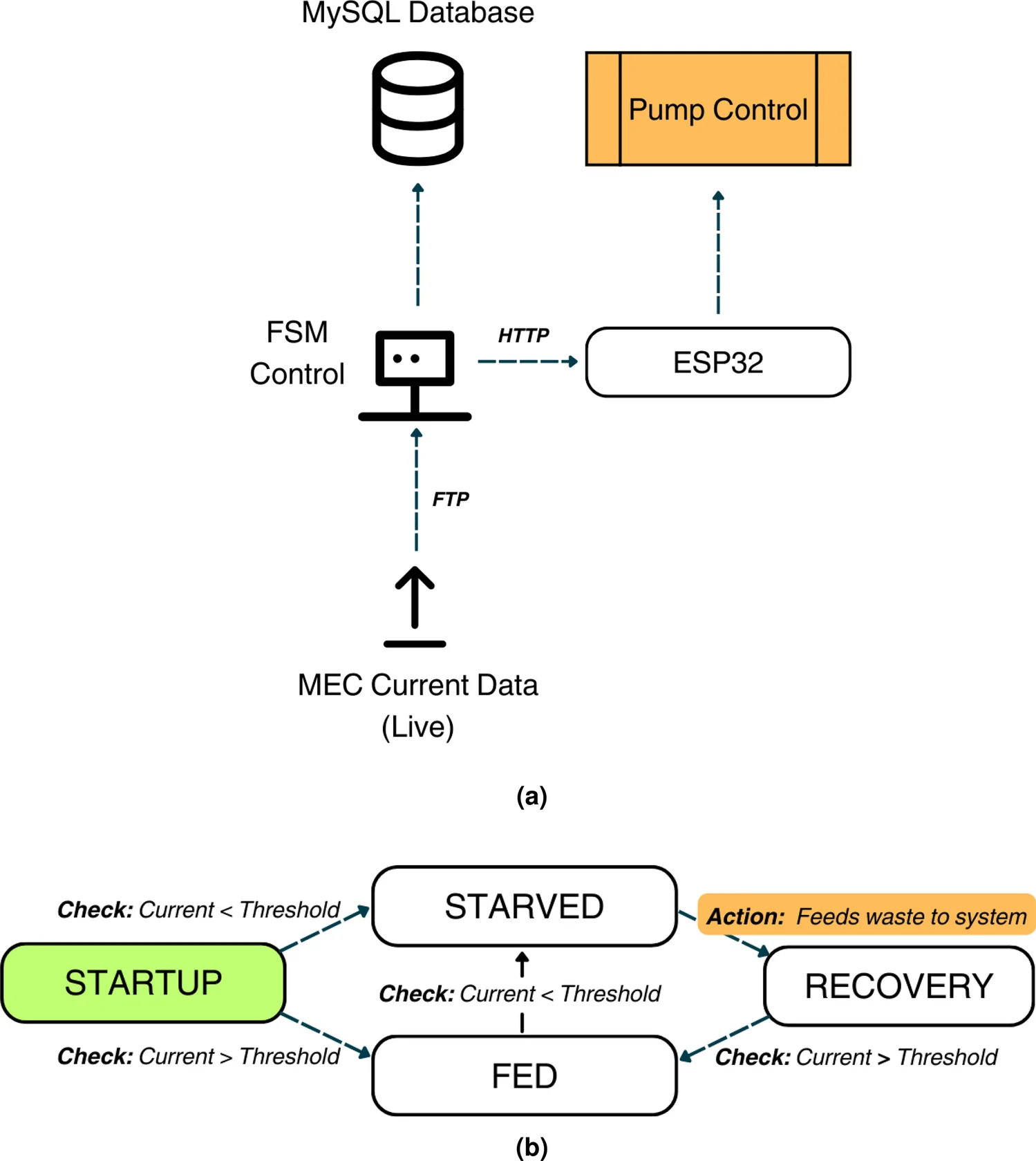
Automated control framework: (**a**) control and data acquisition architecture; (**b**) FSM state logic and transitions.

An overview of the control and data acquisition architecture and FSM logic is provided in Fig. 3.

### Finite state machine development

The operational control of the MEC reactor is modeled through an FSM employing a Mealy machine structure, which encapsulates state transitions and corresponding outputs based on system conditions. The threshold for state transitions within the FSM is determined based on the peak current observed during a typical system dose. Figure 3(b) provides a visual representation of the state transitions and threshold calculations of the FSM. To determine the threshold, the peak current reached after a feeding event was observed during preliminary doses. The threshold was then set to 75% of this peak current value, allowing the system to trigger a new feeding event when the current dropped below this level.

#### Threshold definition

$$I_{\text {peak}} = \max (\text {observed current during example dose period})$$

$$\theta = 0.75 \times I_{\text {peak}}$$

#### FSM definition

$$M = (S, s_0, \Sigma , \delta , \Omega , \gamma )$$

where:

- *S* represents the system states: {Startup, Fed, Starved, Recovery}
- $s_0$ is the initial state: Startup
- *Σ* denotes the input conditions based on the current draw relative to the threshold *θ*: {current draw $\ge \theta$, current draw *< θ*}
- *δ* is the state-transition function defined as $\delta : S \times \Sigma \rightarrow S$
- *Ω* is the finite set of outputs produced by the system
- *γ* represents the output function defined as $\gamma : S \rightarrow \Omega$

### Transition rules

The transition rules defined for the FSM are:

- $$\delta (\text {``Startup''}, \text {current draw }>\theta ) = \text {``Fed''}$$
- $$\delta (\text {``Startup''}, \text {current draw }<\theta ) = \text {``Starved''}$$
- $$\delta (\text {``Recovery''}, \text {current draw }>\theta ) = \text {``Fed''}$$
- $$\delta (\text {``Fed''}, \text {current draw }<\theta ) = \text {``Starved''}$$
- $$\delta (\text {``Starved''}, \text {feed waste action}) = \text {``Recovery''}$$

The threshold parameter *θ* was defined as $0.75 \times I_{\textrm{peak}}$, based on preliminary observations, to trigger feeding once electrochemical activity had sufficiently declined while avoiding premature dosing.

The feeding volume was selected to elicit a measurable current response without overloading the system and was adjusted to explore different operating conditions. These parameters were determined empirically and may require tuning depending on reactor configuration and feedstock. The operational threshold therefore represents a dynamic indicator of microbial activity derived from prior system behaviour rather than a fixed biochemical setpoint.

#### Feedstock characteristics

Molasses was used as the substrate throughout the experiments due to its high soluble chemical oxygen demand (COD) and consistent composition. The molasses used in this study was a cane-derived blackstrap molasses obtained from commercial agricultural feedstock supplies.

Typical composition of cane molasses includes approximately *70%* dry matter, with total sugars comprising around 60–*65%* of the dry matter and a crude protein content of approximately 6–*7%*. Molasses also contains trace minerals and organic nutrients derived from the sugar processing stream. Due to its high fermentable sugar content, molasses rapidly hydrolyses to volatile fatty acids during anaerobic digestion and therefore provides a readily biodegradable carbon source^33^. Molasses was selected as a reproducible model substrate that enables controlled investigation of biosensor-led feeding strategies under stable and well-defined laboratory conditions.

### Offline digestate analysis

After the initial startup period, comprehensive daily analyses of samples were conducted. Each analysis entailed withdrawing 10 mL of digestate from mid-level within the reactor using a silicone tube connected to a syringe via the sample port. Care was taken to ensure the sample accurately reflected the system’s condition. This included thoroughly clearing the feed line before each sample collection.

#### Chemical oxygen demand and soluble chemical oxygen demand

Both Chemical Oxygen Demand (COD) and Soluble Oxygen Demand (sCOD) were measured by a closed reflux colourimetric method using Hach Lange COD Cuvette tests (014, 514 to ISO 6060-1989 DIN 38409-H41-H44). For sCOD, the water sample is centrifuged at 6000 rpm (approx. 2000 RCF) for two minutes to remove the bulk of the suspended solids. The supernatant is pipetted into a clean syringe and filtered through a 0.45 *μ*m pipette filter. The filtered sample is then used as in the tCOD methodology.

#### FOS/TAC

This is a measure used to gauge the balance between acid production and the system’s buffering capacity. Typically, a FOS/TAC ratio above *0.5* can indicate an imbalance between acidogenic and methanogenic biomass; however, there is no consensus in the literature regarding the optimum FOS/TAC ratio, as every system and feedstock combination has a unique optimum ratio^34^. This measurement was carried out using the Nordmann method. In this method, $20 \, \text {mL}$ of fresh sample is typically mixed with $30 \, \text {mL}$ of de-ionized water, and the mixture is titrated using $0.1 \, \text {N}$ sulfuric acid ($\text {H}_2\text {SO}_4$). The mixture is stirred using a magnetic stirrer, and the pH is measured with a calibrated combination pH/temperature probe.

#### Total solids, volatile solids and total suspended solids

Total Solids (TS), Volatile Solids (VS), and Total Suspended Solids (TSS) were measured according to Standard Methods for the Examination of Water and Wastewater^35^. TS was determined by evaporating a known volume of sample in a pre-weighed, dried dish and drying it at 103–105$^\circ$C until constant weight was achieved. VS was measured by igniting the dried TS residue at 550$^\circ$C, with the loss on ignition quantifying the organic solids. TSS was assessed by filtering a well-mixed sample through a pre-weighed filter, drying the filter at 103–105$^\circ$C, and then weighing it to measure the retained solids.

#### pH and conductivity

pH was measured using a benchtop pH meter, with the pH probes calibrated at three points using standard pH buffers. Conductivity was measured using a RS Pro conductivity meter.

#### Gas volume and composition

Gas volume is measured volumetrically using wet tip flow meters. Wet tip flowmeters were calibrated at room temperature using 10% saline solution as the filling liquid. This is to reduce microbial growth and reduce the solubility of biogas in the liquid.

#### Biogas normalisation

Quantification of biogas and methane requires normalisation to account for variations in atmospheric pressure. Atmospheric pressure and temperature are measured with a barometer and temperature probe situated near the volumetric gas flowmeter. Volumes of measured biogas were normalised to NIST Normal Temperature and Pressure (20 $^{\circ }\textrm{C}$, 293.15 $\textrm{K}$, and 101.325 $\textrm{kPa}$)^36^.

#### Biogas composition

Biogas composition was determined by two methods throughout experimentation. Biogas was analysed qualitatively (CH$_4$, CO$_2$, and H$_2$S percentages) by a handheld infrared and electrochemical gas analyser and a dual gas infrared gas sensor was placed in a chamber in line with the gas outlet of the reactor. These sensors detect methane and carbon dioxide between 0–100% (at 0.1% resolution), and propane at 0–2% (0.01% resolution).

### Metric calculations

The average feed interval over the day period was calculated as shown in Equation 1.

1 $$\begin{aligned} f(d) = \frac{1}{|\text {intervals}_d|} \sum _{i \in \text {intervals}_d} i \end{aligned}$$

- *f(d)* : Average feed interval on date *d*.
  - *Definition*: Represents the mean interval duration for feed events on the date *d*.
  - *Purpose*: Analyses the average time between feed events on each day, identifying daily fluctuations.
- $|\text {intervals}_d|$ : Cardinality of the set $\text {intervals}_d$.
  - *Definition*: Total number of feed intervals recorded on date *d*.
  - *Purpose*: Acts as the divisor in the mean calculation, reflecting daily activity levels.
- $\sum _{i \in \text {intervals}_d} i$ : Summation of intervals on date *d*.
  - *Definition*: Sum of all interval durations *i*, where each *i* is the time between consecutive feed events on date *d*.
  - *Purpose*: Aggregates the total time of feed intervals for that day, serving as the numerator in the average interval calculation.
- $\text {intervals}_d$ : Set of interval durations on date *d*.
  - *Definition*: Each *i* in this set is a duration measured between two successive feeding events.
  - *Purpose*: Provides the raw data for analysis, containing specific interval measurements for computation of daily averages.

### Reduced equation for methane yield per gram of COD

The equation to calculate the methane yield per gram of COD added has been listed under Equation 2.

2 $$\begin{aligned} Y = \frac{B \times p}{C \times \overline{f}(d)} \end{aligned}$$

where *Y* represents the yield of methane per gram of COD added, linking operational input variables to the efficiency of methane production.

### Key variables

- *C*: COD mass per feed (g) = $\text {feedstrength} \times \text {feed volume}$
- $\overline{f}(d)$: Average number of feeds per day
- *B*: Daily biogas flowrate (NL).
- *p*: Proportion of methane in biogas (typically 0.55).

### Statistical analysis

Correlation analysis was performed to assess the relationship between reactor current (currentA) and other measured variables using Pearson’s product–moment correlation coefficient.

Correlations were evaluated using daily averages, 7-day fixed averages, and 7-day moving averages to examine relationships across different temporal resolutions. Shorter windows preserve operational variability, while longer averaging reduces noise and highlights underlying trends in biological time-series data. P-values were calculated based on a t-distribution under the assumption of bivariate normality. All analyses were performed using the SciPy library in Python.

## Results

### Use of FSM-controlled feeding for system startup

**Fig. 4:**
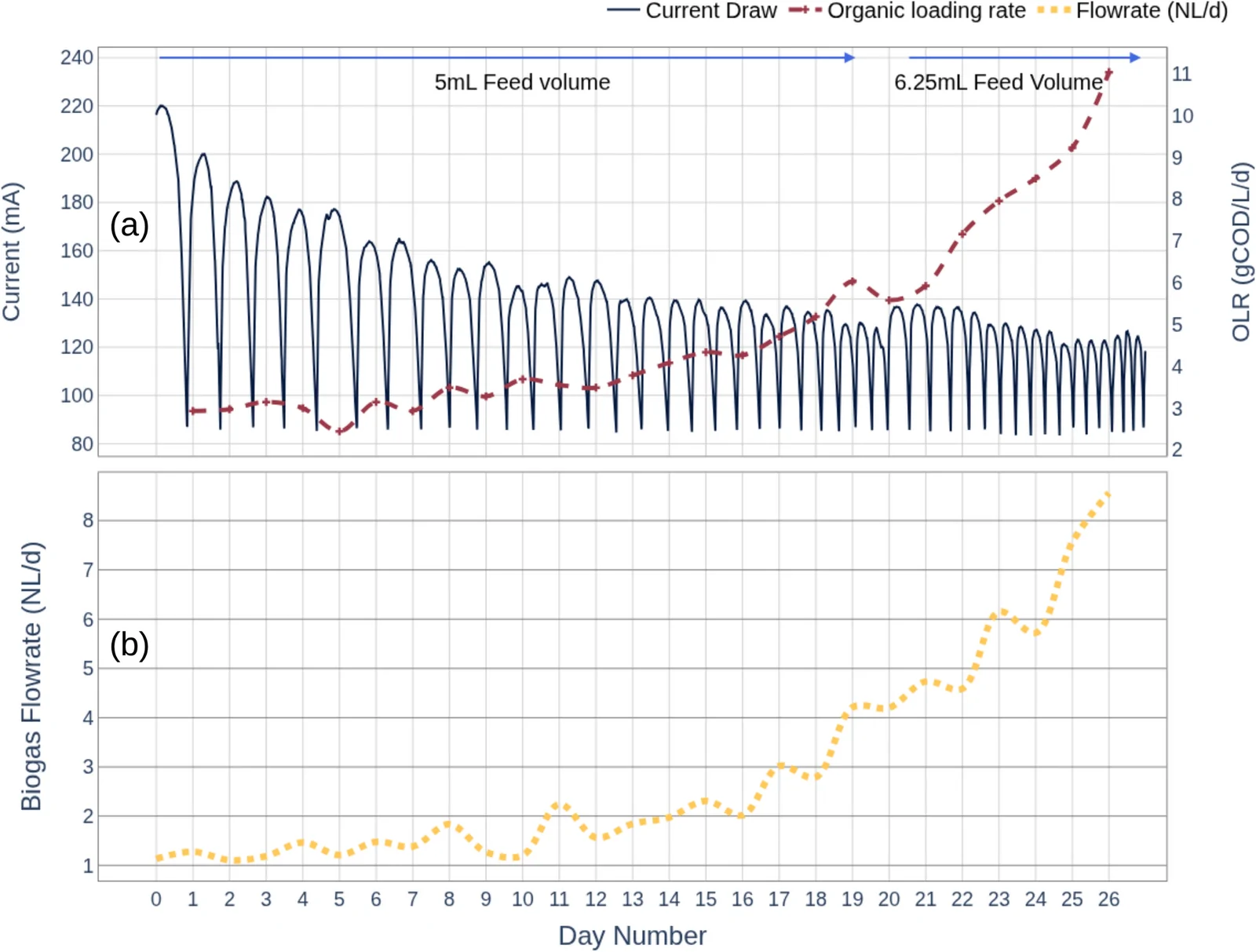
Startup loading ramp under FSM-controlled feeding. (**a**) Current response and calculated organic loading rate (OLR) during startup. Feed events were identified via peak detection on inverted current signals. A step increase in feed volume from 5 mL to 6.25 mL is indicated. (**b**) Monitored biogas flowrate over the corresponding period.

FSM-controlled feeding was implemented from the beginning of the experiment. During this phase, pure molasses (5–6.25 mL, typical COD value: 950 gCOD L$^{-1}$) was added to the system. Figure 4 shows the current draw data over this startup period. By inverting the current and applying peak detection, the feed event times were extracted. OLR was calculated based on the average feed interval, which was used to estimate the number of feed events occurring over a 24-hour period, as detailed in Section “Metric calculations”.

**Fig. 5:**
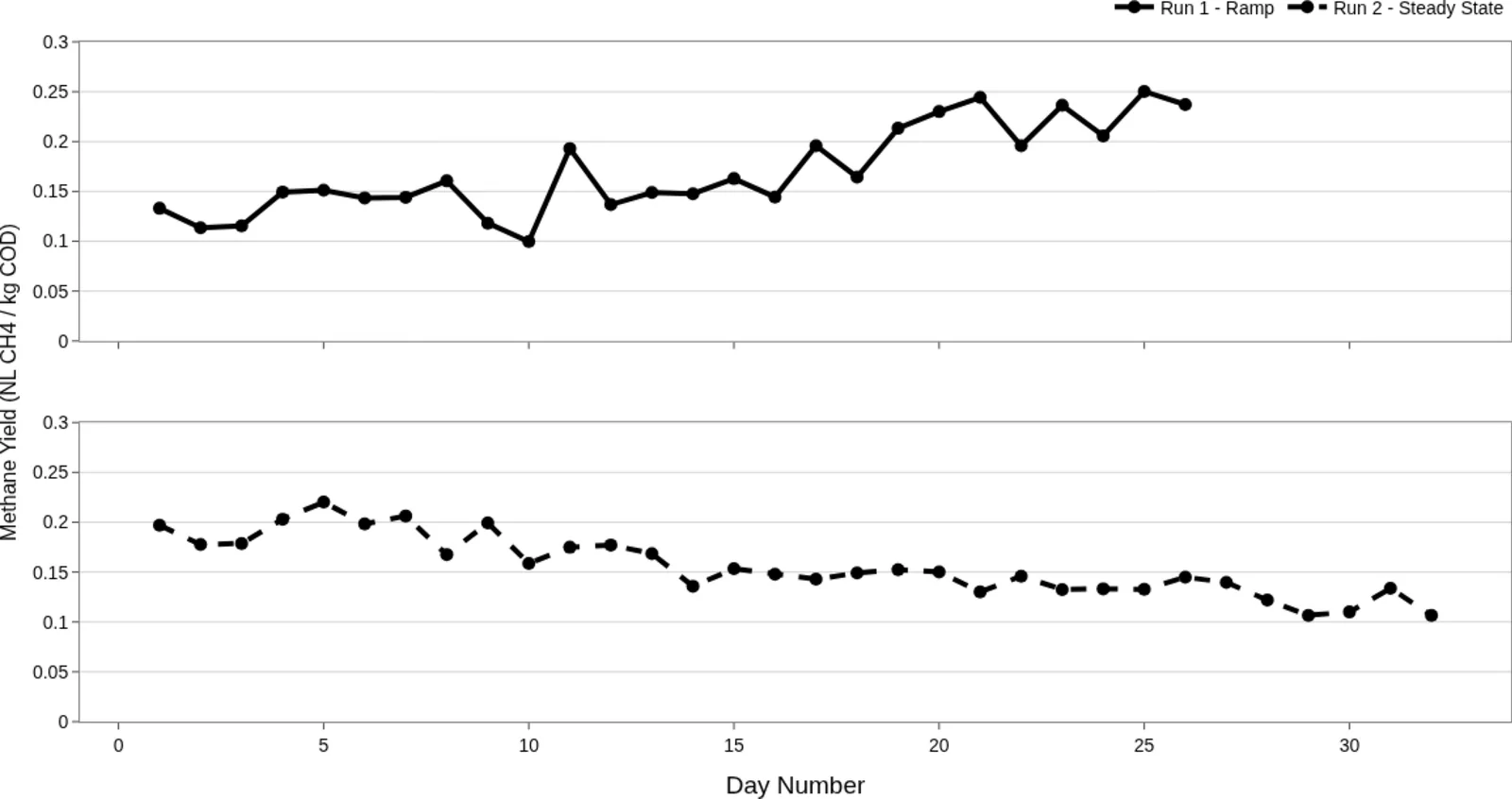
Apparent methane yield relative to COD dose during startup and steady-state operation. (**a**) Apparent methane yield during Run 1 startup operation under FSM-controlled feeding. (**b**) Apparent methane yield during Run 2 steady-state operation following recovery from disturbance. Each yield value reflects methane production relative to the COD dose added through FSM-controlled feeding, rather than a complete COD conversion efficiency.

Apparent methane yield trends based on COD added for Runs 1 and 2 are shown in Fig. 5. During startup operation, the apparent methane yield increased over time, indicating progressive development of methanogenic activity under FSM-controlled feeding. This trend occurred alongside an increase in the calculated OLR over the duration of the test. On day 19, the feed volume was increased from $5\,\textrm{mL}$ to $6.25\,\textrm{mL}$ of molasses, increasing the feed dose relative to the reactor working volume from 2.37 to $2.9\,\mathrm {gCOD\,L^{-1}}$. Throughout the startup period, the peak current amplitude progressively decreased and approached a plateau toward the end of the test interval.

The interval between feed events was derived from the module’s current response data. After each feed event, the current increased. This rise in current is consistent with increased electrochemical activity following substrate addition and is interpreted here as reflecting enhanced electrogenic microbial metabolism. The response to the increased feed volume on day 19 is evident in the daily average feed interval. The change in feed increased the amount of organic matter in the system, which lengthened the time available for microbial metabolism and thus extended the period needed to fully break down the organic material. The increase in feed volume introduced a greater organic load to the system. The longer interval before the subsequent current decline suggests an extended processing period under the applied control logic. This behaviour is consistent with prolonged microbial substrate utilisation, although microbial activity was inferred from current response rather than directly measured. As a result, the decline in current draw occurred later, reflecting a delay in the system’s readiness for the next feeding cycle. Over the following five days, the calculated biosensor-led OLR progressively increased under the FSM control framework. This trend suggests acclimation to the increased feed volume under the tested conditions.

Over the course of the test period, biogas production gradually increased, reaching an apparent methane yield of approximately $0.25 \, \mathrm {NL\ CH_4\,gCOD^{-1}_{added}}$. This value is below the theoretical maximum of approximately $0.375 \, \mathrm {NL\ CH_4\,gCOD^{-1}}$ expected for complete COD conversion to methane under the gas normalisation conditions used in this study at 20 $^\circ$C and 1 atm. This difference may be attributed to incomplete substrate conversion, biomass growth, residual soluble and particulate organics, and potential losses associated with lab-scale continuous operation. Because yield was calculated from daily methane production and daily influent COD loading, these values are interpreted as apparent daily yields rather than complete COD conversion efficiencies. Despite this, the observed yields indicate that the system maintained functional methanogenic activity under FSM-controlled feeding.

### Monitoring offline parameters and current draw during steady state operation

**Fig. 6:**
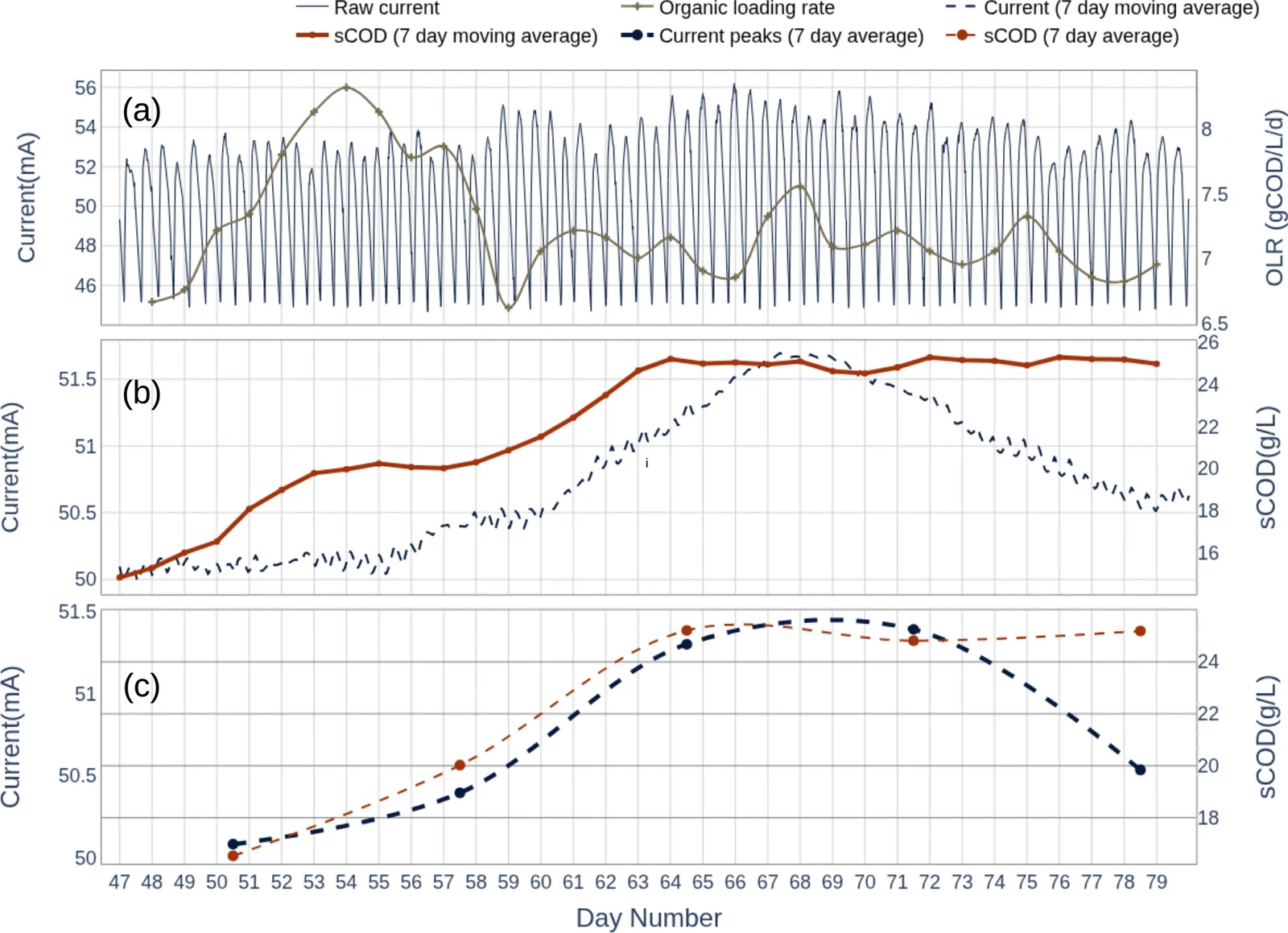
Steady-state operation following power-outage recovery. (**a**) Raw current response with inferred organic loading rate (OLR), indicating stable loading conditions. (**b**) Raw current (7-day moving average) alongside soluble COD (sCOD, 7-day moving average). (**c**) 7 day fixed-interval averages of current peaks and sCOD.

After the initial 24 days, the system encountered a power outage, leading to the malfunction of a pump. Consequently, this triggered a starvation period, resulting in a substantial decrease in current, falling well below the pre-set operational threshold for the experiment. This reduction significantly altered the system’s performance regarding the maximum current the electrode could attain under the established feeding strategy. This is indicated by the lower current response seen in Fig. 6 when compared with Fig. 4. In reaction to starvation, feeding was manually initiated, eliciting a current response, although the peak current remained lower than before. This preliminary response facilitated the adjustment of the operational threshold to 45 mA, as documented in the current range shown in Fig. 6. This adjustment demonstrates that the FSM threshold was dependent on the previously observed current range and required recalibration following disturbance. The need for manual threshold modification highlights a limitation of a fixed current-threshold control strategy when system history or biofilm activity has changed. Once feed events were resumed, the current signal returned to a repeatable oscillatory pattern consistent with automated FSM operation, but within a reduced current range relative to pre-disturbance conditions. This repeatable behaviour was also reflected in the relationship between current and sCOD concentration, where Pearson correlation analysis showed a moderate positive correlation for daily averages (*r = 0.61*, $p = 1.6 \times 10^{-4}$), and stronger correlations for longer averaging periods, with *r = 0.80* ($p = 1.0 \times 10^{-1}$) for the 7 day average and *r = 0.82* ($p = 4.4 \times 10^{-9}$) for the 7 day moving average. Throughout this phase, the OLR (inversely proportional to feeding interval) remained relatively stable, with an average of 7.3 $\mathrm {gCOD\,L^{-1}\,d^{-1}}$ and a coefficient of variation of 5.9%.

### Use of FSM-controlled feeding under varying feed strength conditions

Run 3 was conducted as a separate experimental phase using two reactors operated in parallel. These reactors were not continuations of the single reactor system used in Runs 1 and 2. Instead, they were established as independent MEC-AD reactors using the same reactor design, electrode configuration, inoculum source, feedstock, and FSM control architecture. Prior to the monitored feed strength variation period, both reactors underwent a 36-day stabilisation phase under FSM-controlled feeding to establish repeatable current responses and stable operating conditions.

In this phase a binary feeding method was introduced, where water and molasses were supplied separately into the system. This setup was crucial for enabling feed dilution and inducing controlled digestate washout, which is essential to produce steady state conditions and allow the response to a controlled input to be monitored. The primary objective was to observe biosensor and control system responses relative to the varying strength of input feedstock.

**Fig. 7:**
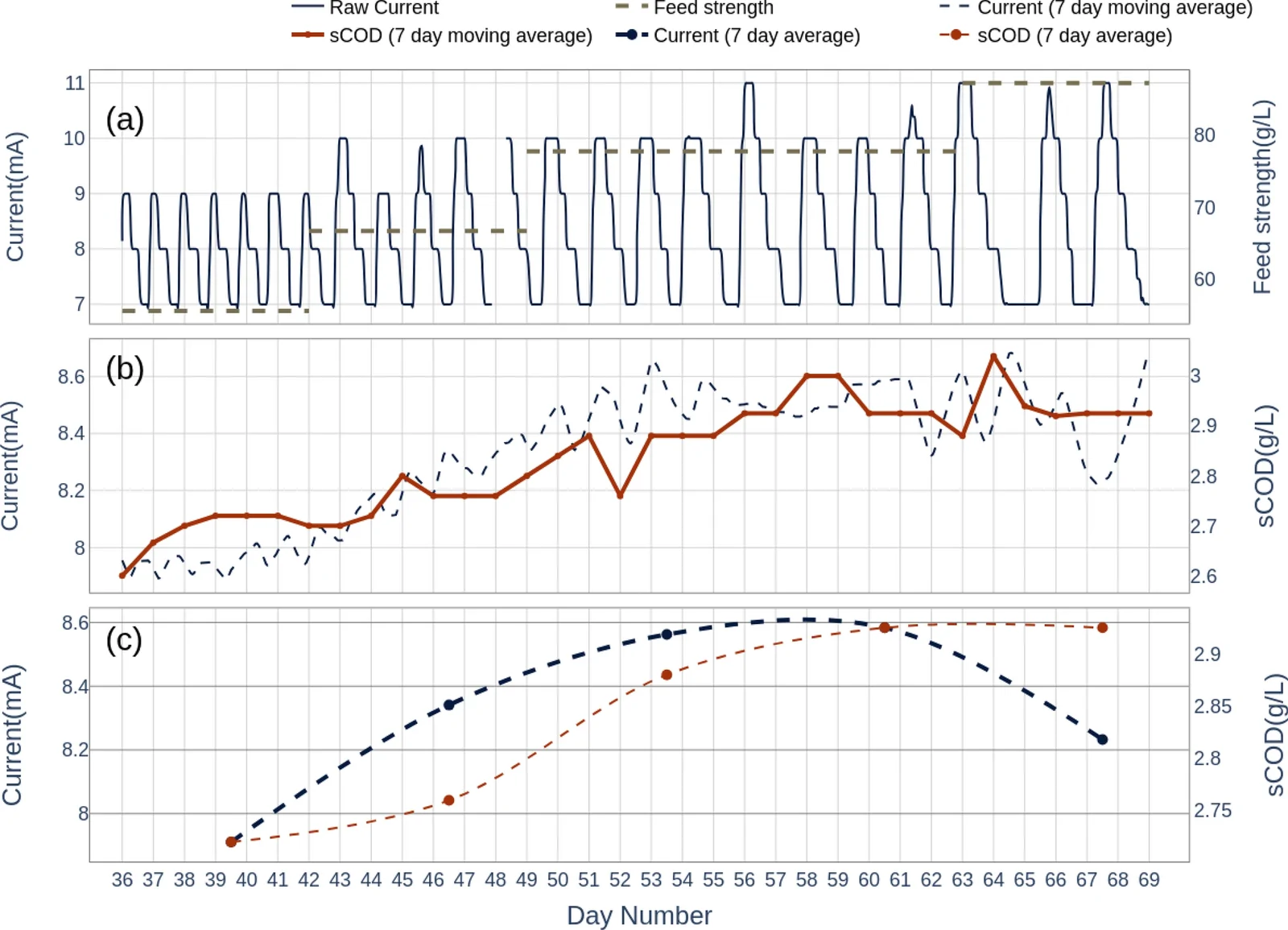
Biosensor response to varying feed strength in Reactor 1. (**a**) Raw current response with corresponding feed strength variation, showing discrete step changes in feed concentration. (**b**) Current (7-day moving average) and reactor sCOD (7-day moving average) during the feed strength variation period. (**c**) 7-day fixed-interval averages of current and reactor sCOD.

**Fig. 8:**
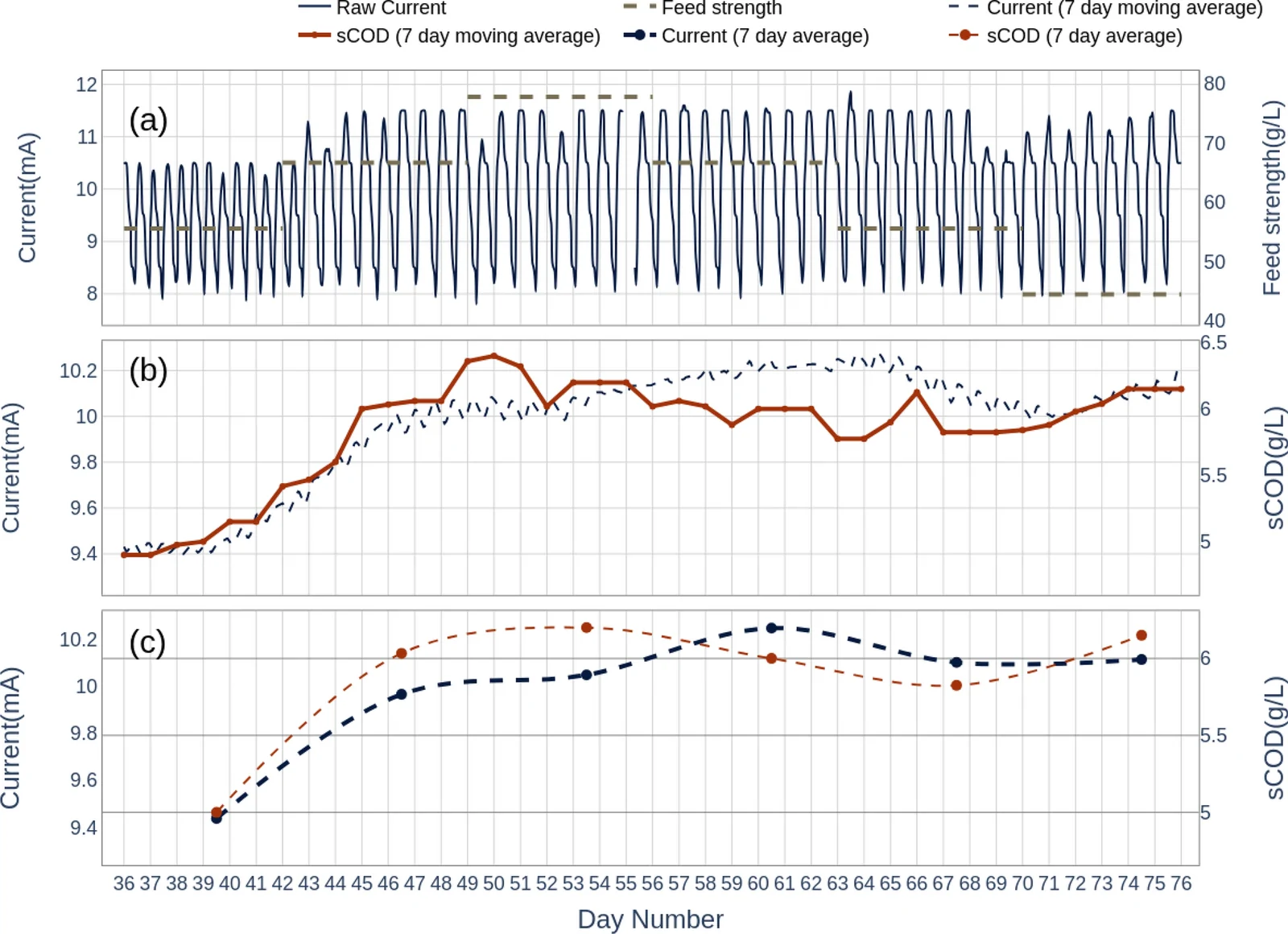
Biosensor response to varying feed strength in Reactor 2. (**a**) Raw current response with corresponding feed strength variation, showing discrete step changes in feed concentration. (**b**) Current (7-day moving average) and reactor sCOD (7-day moving average) during the feed strength variation period. (**c**) 7-day fixed-interval averages of current and reactor sCOD.

**Fig. 9:**
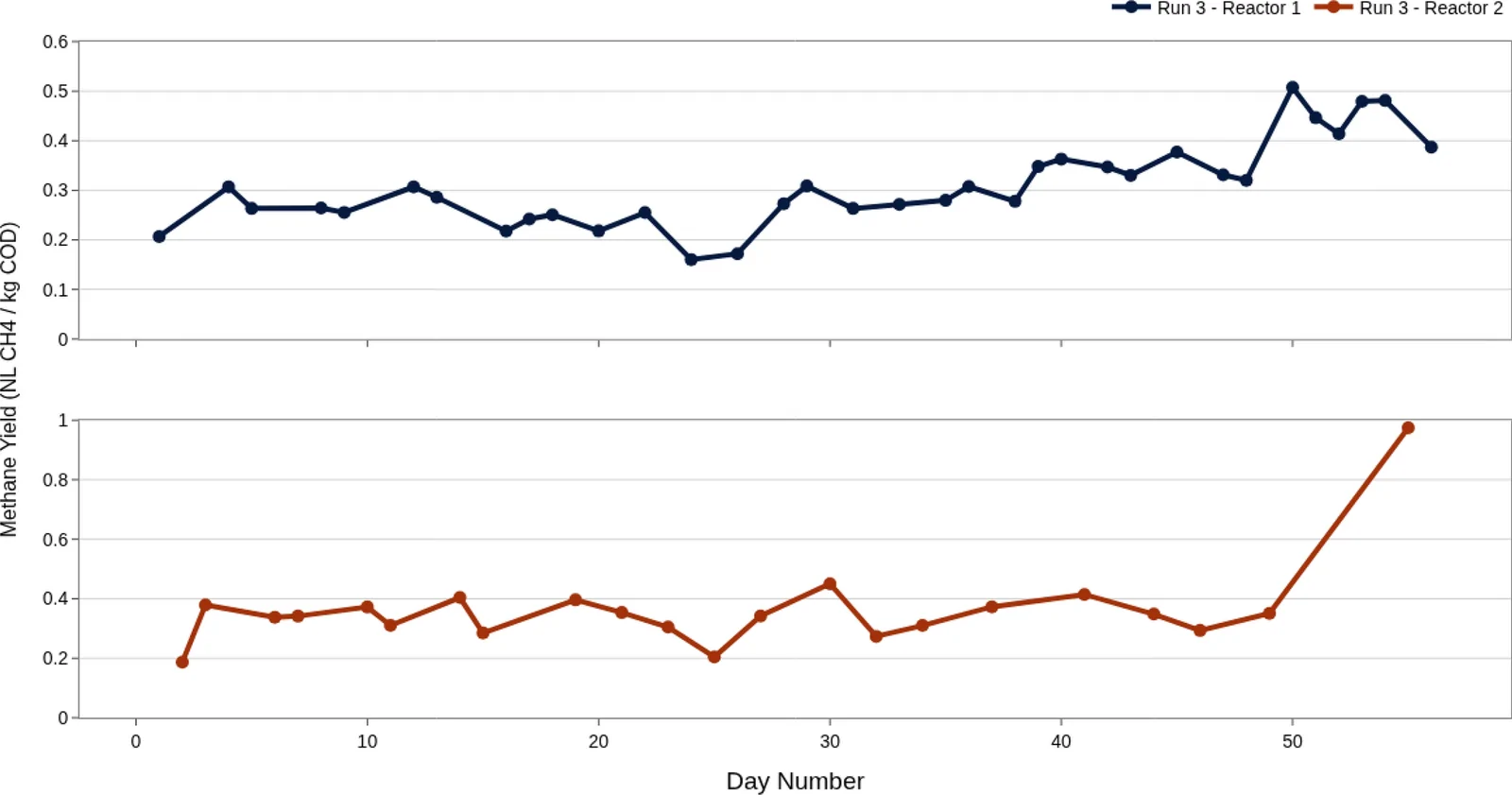
Apparent methane yield relative to COD dose during feed-strength variation experiments. (**a**) Apparent methane yield in Reactor 1 during varying feed-strength operation. (**b**) Apparent methane yield in Reactor 2 during varying feed-strength operation. Each yield value reflects methane production relative to the COD dose added through FSM-controlled feeding, rather than a complete COD conversion efficiency. Elevated apparent yields during extended feed intervals may reflect continued methane production from residual organics retained within the reactor.

Apparent methane yield trends based on COD added during the feed-strength variation experiments are shown in Fig. 9. The calculated yields provide additional context for interpreting the current response, showing that methane production was maintained during the feed-strength variation period. Variability in apparent yield reflects the dynamic feeding pattern and the influence of residual organics retained within the reactor under extended hydraulic retention conditions.

Statistical analysis of offline parameters during this phase for both reactors further supports the observed relationship between sCOD and module current draw, as detailed in Table 2. Reactor 1 exhibited significant daily and moving-average correlations, with a daily average correlation of *R = 0.75*, $p = 3.4 \times 10^{-5}$, and a 7-day moving-average correlation of *R = 0.86*, $p = 8.1 \times 10^{-11}$. Reactor 2 also showed improved correlation after temporal smoothing, with a 7-day moving-average correlation of *R = 0.86*, $p = 4.2 \times 10^{-13}$, compared with the daily average correlation of *R = 0.38*, $p = 4.0 \times 10^{-2}$. These statistical findings demonstrate a positive correlation between sCOD and module current draw under the tested operating conditions. While this association indicates co-variation, it does not establish fixed causality. The results suggest that current may serve as a proxy for soluble organics during stable, uninhibited operation.

**Table 2:** Pearson Correlation Coefficients for sCOD in MEC-AD Reactors.

**Reactor**	**Period**	**Coeff.**	**P-val**
R1	Daily Average	0.75	$3.4 \times 10^{-5}$
R2	Daily Average	0.38	$4.0 \times 10^{-2}$
R1	7 Day Average	0.68	$2.1 \times 10^{-1}$
R2	7 Day Average	0.89	$2.0 \times 10^{-2}$
R1	7 Day Moving Average	0.86	$8.1 \times 10^{-11}$
R2	7 Day Moving Average	0.86	$4.2 \times 10^{-13}$

The feed strength variation for Reactor 1 lasted 33 days, concentrations of the feed supplied to the reactors were varied between 55 g/L and 88 g/L with weekly adjustments as detailed in Fig. 7. A consistent feed strength of 78 g/L was maintained for two weeks to assess potential lagged current responses due to organic material accumulation, considering the impact of prolonged hydraulic retention relative to the test duration. Using an approximation of one feed per day results in a hydraulic retention time (HRT) of approximately 36 days. As the set feed strength was increased, an increase in sCOD was observed, corresponding to an increase in current response. Reactor 2 achieved operational stability before Reactor 1, leading to an earlier initiation of feed variation in Reactor 2 by one week. In Reactor 2, as detailed in Fig. 8, the feed strength varied from 45 g/L to 78 g/L across weekly intervals. An initial increase in sCOD was noted, however reducing the feed strength can be seen to have a less defined impact on current response compared to an increase. This could be a result of the high HRT relative to the test period. This aligns with a maintained sCOD concentration over the period when feed strengths were reduced. Testing for extended periods with reduced feed strength may yield clearer results on how the current responds to reducing sCOD levels in the digestate.

### Observations from monitoring online data

**Fig. 10:**
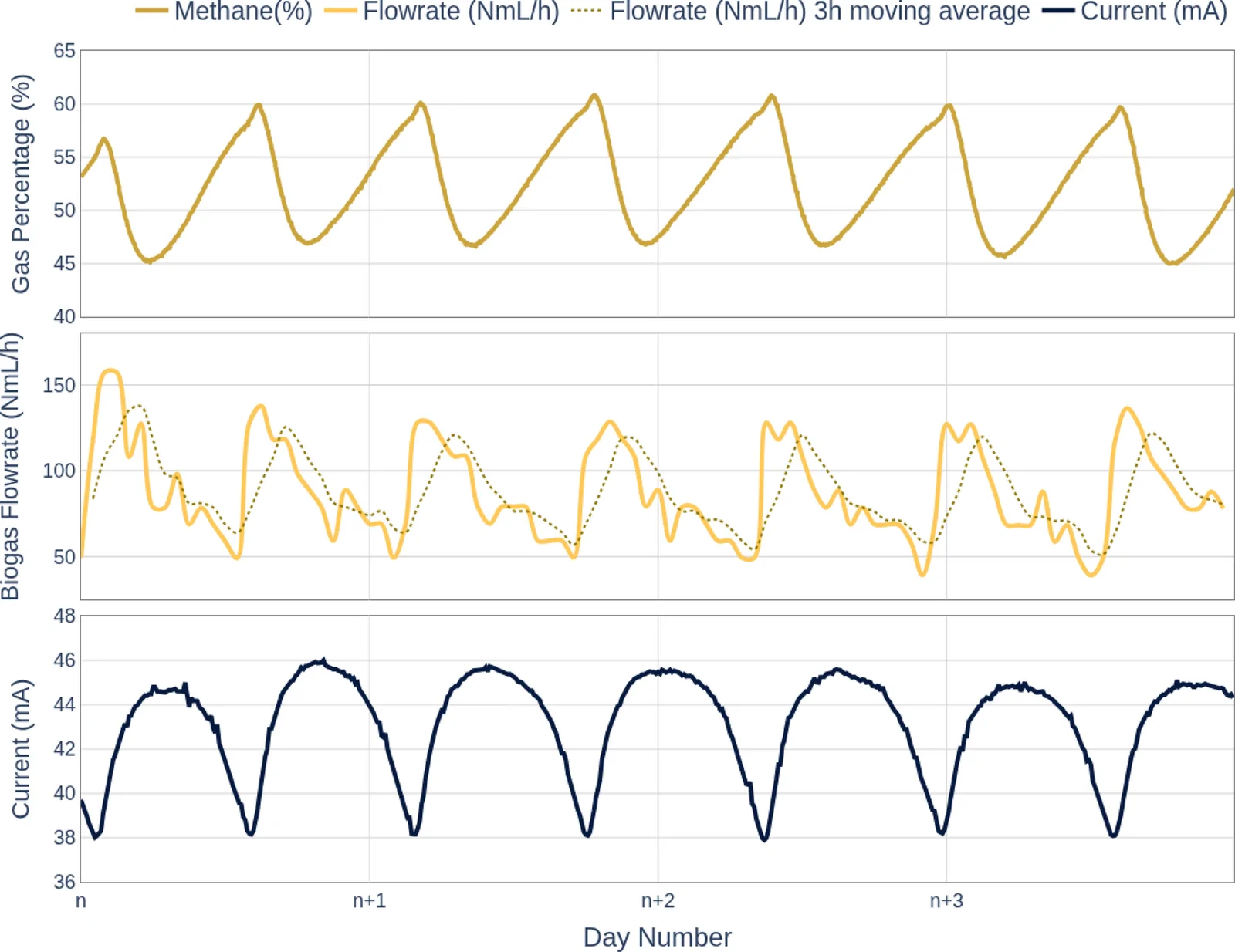
Steady state operation detailed in Section "Monitoring offline parameters and current draw during steady state operation".

Methane concentration in the biogas consistently varied between 45% and 60%, indicating stable methanogenic activity and reaffirming the effectiveness of the FSM-controlled feeding protocol. Peaks in biogas flow rate typically followed these feed inputs, showcasing an immediate microbial response to substrate availability, enhanced by a three-hour moving average to reduce data noise. The observed sinusoidal fluctuations in current draw reflect variations in the metabolic rates of electroactive bacteria, closely aligning with measured biogas production. This synchronisation underscores the FSM’s capability to precisely coordinate feed inputs with microbial activity. Representative steady-state operation is shown in Fig. 10, while additional time-series visualisations for the feed-strength variation experiments are provided in the Supplementary Information.

### FSM response failure and recovery strategies

**Fig. 11:**
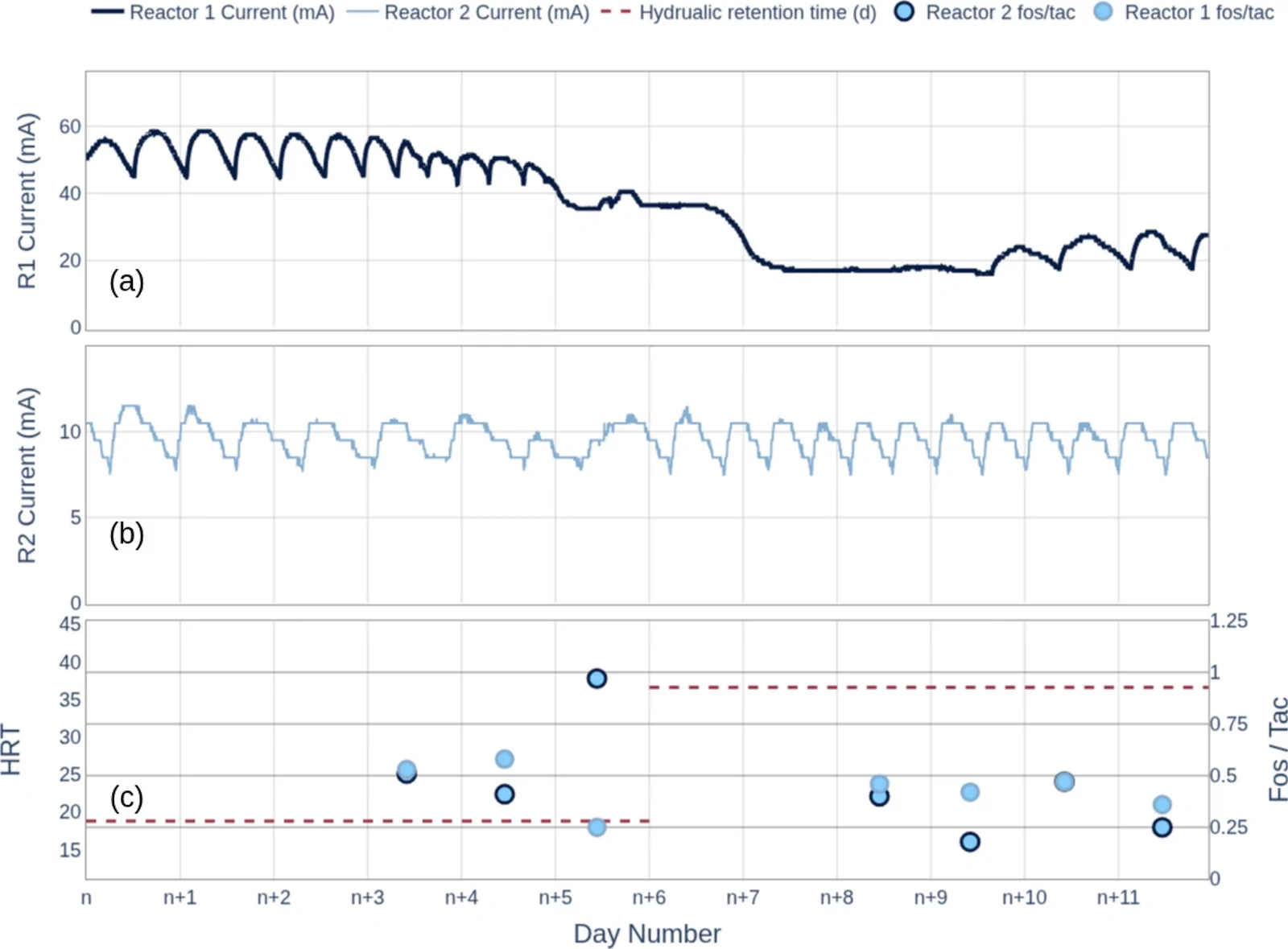
Temporal dynamics of electrical current, HRT, and FOS/TAC ratios in Reactors 1 and 2 during the start-up phase prior to feed strength variation. (**a**) Reactor 1 current response showing a decline and failure to exit the recovery state on day 5, with recovery of automated control observed after day 9 following adjustments to HRT and the control threshold. (**b**) Reactor 2 current response showing maintained operation throughout the period, with more consistent responses observed from day 6 onward following an increase in HRT. (**c**) HRT and FOS/TAC ratios for both reactors, highlighting the correlation between a spike in Reactor 1 FOS/TAC and the failed current response. HRT was subsequently increased to counteract biomass washout.

Figure 11 illustrates an incident where the FSM failed to elicit a response from the current, consequently leaving the reactor in a recovery state. To restore operational status, it is critical to assess the reactor’s health by examining metrics such as the FOS/TAC ratio, which details the balance of VFA to buffer. If this ratio significantly exceeds the normal range, it is essential to allow adequate time for the reactor to achieve stability before resuming operations^37^. Once stability is achieved, the current threshold is recalibrated to the newly stabilised current range, and feeding events can be resumed. The top two graphs in Fig. 11 display the module current responses of Reactor 1 and Reactor 2 over 12-days around the startup period, during which diluted molasses was supplied to induce washout conditions and achieve steady-state operation. Reactor 1 shows a failure in current response on day 5; as a result, the FSM did not transition out of the recovery state. This failure coincided with a sharp spike in the FOS/TAC ratio, clearly signaling that operational changes were needed to eliminate washout issues. To rein in biomass washout, the HRT was increased and mixing intensity was deliberately reduced.

While increases in current were observed to coincide with increases in biogas flowrate following feeding events (Fig. 10 and Supplementary Figures S1–S2), this does not imply a direct or consistent relationship between these parameters. Over short, stable operating periods, both signals respond to substrate addition and may appear correlated. However, as shown in Fig. 11, operational disturbances can shift the current response range independently of overall system performance. This indicates that current should be interpreted as an indirect indicator of system activity rather than a direct proxy for biogas production.

**Table 3:** Summary of experimental runs used to evaluate FSM-controlled feeding.

**Run**	**Duration**	**Reactor(s)**	**Objective and key conditions**
Run 1: Startup (No Washout)	0–26 days	Single reactor	Evaluate FSM ramp-up during early operation. Pure molasses feed (5–6.25 mL); no washout.
Run 2: Post-Disturbance Steady State	47–79 days	Same reactor (restart)	Assess recovery and stability after starvation. Threshold recalibration; no washout; increased digestate solids.
Run 3: Independent Parallel-Reactor Washout and Feed Variation	Stabilisation ($36\,\textrm{d}$) + monitored phase: R1 (36–69), R2 (36–76)	Independent Reactor 1 and Reactor 2	Evaluate FSM under washout and varying feed strength. Reactors were started as independent MEC-AD systems using the same reactor design, inoculum source, feedstock, and FSM control architecture. Binary feed (water + molasses) and controlled dilution were applied following stabilisation.

## Discussion

### Interpretation of startup behaviour and peak current attenuation

During the startup phase, a gradual reduction in peak current amplitude was observed despite increasing organic loading rates. This attenuation in peak response may be attributed to changes in microbial community structure and biofilm development dynamics, however microbial composition and biofilm structure were not directly characterised in this study. The following mechanisms are therefore proposed as plausible explanations based on existing literature. As substrate availability increases, planktonic microbial populations may expand alongside electrode-associated communities. These non-electrode-interactive microorganisms may compete for available soluble organics, potentially reducing the fraction of substrate directed toward electrogenic metabolism at the anode^38,39^. Planktonic biomass may vary due to growth on residual organics and biofilm detachment under mixing. As the fraction of influent COD converted to current was not directly quantified, the role of planktonic consumption in peak-current attenuation is proposed as a plausible mechanism rather than a confirmed cause.

In addition, increasing biofilm thickness during acclimation can introduce mass transfer limitations at the electrode surface. Diffusion constraints may restrict substrate access to electroactive bacteria located deeper within the biofilm matrix, thereby limiting electron transfer efficiency. Fouling due to biomass accumulation or extracellular polymeric substances (EPS) may further contribute to increased internal resistance. Together, these mechanisms provide a plausible explanation for the reduced peak current amplitude observed during ramp-up.

The increase in feed volume on day 19 resulted in longer feeding intervals and sustained elevation of current following each dosing event. This adjustment offers valuable insights into the system’s response to variations in organic load. This behaviour is consistent with an increased metabolic processing time associated with higher substrate loading. Over subsequent days, the biosensor-controlled organic loading rate progressively increased, suggesting system acclimation to the elevated feed strength. Startup phases in MEC-AD systems are often underreported and can vary substantially depending on reactor architecture and operational conditions^40,41^. The behaviour observed here likely reflects a stabilisation period during which electroactive biofilms matured and adapted to the applied loading conditions^42,43^.

The FSM-based control strategy may facilitate dynamic ramp-up by adjusting feeding intervals in response to microbial activity. Although offline reactor characterisation was not conducted during early startup, it is plausible that biomass growth and increasing electroactive population density contributed to the progressive rise in calculated organic loading rate^44^.

### Current draw as a proxy for soluble organics: strength and limitations

Correlation analysis demonstrated a consistent relationship between module current draw and soluble chemical oxygen demand (sCOD) across daily and moving-average datasets. Stronger correlations were observed when using seven-day moving averages, suggesting that short-term sampling variability and operational noise can obscure underlying biochemical trends. These results describe statistical co-variation under the tested conditions and do not establish direct causality between current and COD removal.

These findings support the hypothesis that electrode current can serve as an indirect indicator of soluble organic availability within the reactor. As electrogenic microorganisms metabolise substrates, electron transfer to the anode generates measurable current, linking electrochemical response to substrate degradation dynamics. The consistent statistical association across averaging methods reinforces the validity of current as a functional biosensor for soluble organics during stable and uninhibited operation.

A key limitation of this approach is that the current signal may become non-representative of overall digestion performance during inhibitory or imbalanced states. In inhibited states, soluble organics may accumulate without corresponding increases in current generation^45,46^. Examples include inhibition of methanogenesis, substrate overloading, or accumulation of volatile fatty acids that suppress downstream biological activity without proportionally affecting anodic electron transfer. Under such conditions, the decoupling of current from sCOD could lead to misleading interpretations if current is used as a sole control variable^47^. In the context of FSM-based control, such decoupling may result in inappropriate feeding decisions if threshold triggers rely exclusively on current behaviour. Therefore, while current draw is a valuable indicator of microbial activity, it should be interpreted within the broader biochemical context of reactor health and, where possible, complemented by additional monitoring parameters.

### Impact of disturbances on current response and control stability

The system’s response to pump failure and temporary starvation highlights the sensitivity of electroactive biofilms to operational disturbances. A marked reduction in peak current and operational threshold was observed following downtime. Even brief starvation periods may significantly affect biofilm activity, potentially reducing electroactive population density or altering biofilm structure. Because the FSM uses a threshold derived from prior current responses, starvation events can shift the baseline and peak current values, reducing the reliability of a static threshold and necessitating recalibration. Extended starvation can result in cell death within deeper biofilm layers, leading to accumulation of inactive biomass and EPS. Such layers may increase resistance and reduce effective electron transfer, diminishing peak current response. The altered current profile following disturbance suggests that the electrode signal is sensitive not only to substrate availability but also to biofilm integrity.

This sensitivity presents both opportunities and challenges. On one hand, abrupt deviations in current response may serve as diagnostic indicators of hardware failure or biological instability. On the other hand, reliance solely on current thresholds for control may reduce robustness in the presence of transient disturbances. Adaptive threshold recalibration or integration of additional biochemical indicators may therefore improve system resilience.

### Response to feed strength variation and the role of HRT

Variations in feed strength produced measurable changes in both sCOD concentration and current response. Increases in substrate concentration were associated with elevated sCOD and corresponding increases in current, consistent with enhanced microbial metabolic activity. However, reductions in feed strength produced less pronounced current responses, particularly under conditions of extended hydraulic retention time (HRT).

The relatively long HRT (approximately 36 days under typical feeding frequency) may have dampened short-term reductions in substrate concentration, maintaining residual sCOD levels within the reactor. This buffering effect likely reduced the magnitude of observable current decreases following feed dilution. Extended observation periods under reduced loading conditions would be required to fully characterise the temporal relationship between decreasing sCOD and electrochemical response.

Comparable studies have reported similar relationships between dosing events, current spikes, and organic degradation^48^. The present findings reinforce the interdependence between substrate strength, soluble organics, and electroactive microbial metabolism. Nevertheless, system dynamics are influenced by retention time, microbial adaptation, and mass transfer effects, all of which must be considered when interpreting biosensor outputs.

### Implications for biosensor-led control and system development

Through the integration of live methane monitoring and FSM control, this study demonstrates the potential of online monitoring for regulating feed events within an MEC-AD system. The results show that electrode current provides meaningful real-time insight into microbial activity and soluble substrate dynamics, enabling adaptive feeding during startup, steady-state operation, and feed-strength variation. The observed stability in methane concentration, coupled with pronounced fluctuations in biogas flowrate synchronised with FSM-initiated feeding events, further reflects the responsiveness of the control strategy.

The measured electrical current was used in this study as a process-control signal rather than as a direct measure of methane-forming electron flux. Any methane-equivalent contribution estimated from current would therefore represent a theoretical upper bound based on complete conversion of electrical current to methane, rather than an experimentally measured contribution. Since current-to-methane Faradaic efficiency was not independently quantified in this system, such an estimate should be interpreted only as an order-of-magnitude comparison. This comparison is useful for assessing the relative scale of the electrical input compared with the measured methane flow, but it should not be interpreted as evidence of direct electrochemical methane production.

However, effective implementation requires consideration of biofilm sensitivity, disturbance recovery, and potential biochemical decoupling under inhibitory conditions. The integration of complementary sensing modalities, such as alkalinity monitoring, FOS/TAC ratios, or gas composition analysis, may enhance robustness and reduce reliance on a single electrochemical parameter. Future system development should therefore focus on fault detection, adaptive thresholding, and multi-sensor integration to improve resilience under variable operational conditions. Since validation was limited to 2 L single-chamber reactors operated with a controlled molasses substrate, further studies using larger-scale systems and more complex, heterogeneous waste streams are required to assess broader robustness and practical applicability.

### Study limitations

Several limitations should be acknowledged. The experiments were conducted using molasses as a relatively simple and readily biodegradable substrate. More complex feedstocks may introduce variable hydrolysis kinetics and conductivity fluctuations that could influence current response characteristics. In addition, correlation analysis was based on averaged datasets, and higher-frequency biochemical monitoring may reveal additional transient dynamics.

Despite these limitations, the present study demonstrates the feasibility of utilising electrode current as a biosensor for adaptive feeding control in MEC-AD systems and provides a foundation for further development toward scalable and robust automated operation. A further limitation is that the FSM relies on current thresholds derived from prior operating behaviour. Following disturbances such as starvation, the achievable current range may change, requiring manual recalibration of thresholds for continued operation. In addition, current may become decoupled from COD removal under inhibitory conditions, meaning that current alone may be insufficient for reliable control without additional monitoring inputs.

## Conclusions

This study demonstrates the feasibility of an FSM for real-time automated feeding control in lab-scale MEC-AD systems. The integration of this control method improved operational consistency under the tested laboratory conditions and indicates potential for reduced manual intervention, subject to further validation at larger scale. Prior work has highlighted the infancy of using MEC signals for process monitoring^49^ and the necessity to draw from fields like computer science to enhance capabilities in signal interpretation and control and materials engineering to overcome limitations faced in module development^10^. This research adopts an interdisciplinary approach that bridges automation with microbial electrochemistry. Using software-based control to interface with microcontroller hardware to trigger dosing pumps, we develop a practical control system for effective data integration, thereby enhancing the implementation of biosensor-led feeding control in MEC-AD systems.

1. **Investigate the Impact of System Downtime and Current Recovery** As noted in Section "Monitoring offline parameters and current draw during steady state operation", the starvation period induced by power loss to the pump resulted in a decreased current draw. Upon resuming stable operation, the current draw from the MEC system was notably lower than previously observed. This reduction is thought to be related to biofouling, as suggested by literature assessments. To enhance the reliability and responsiveness of MECs in continuous operation, further investigation into the impact of system downtime is essential. A better understanding of the causes of the current drop will help inform the design of effective recovery strategies.
  Furthermore, it is probable that the signals from the MEC will require normalisation relative to the given response range. Focusing on relative trends rather than absolute values is crucial, especially in the context of system downtime. This approach draws parallels with continuous glucose monitoring systems (CGMS) in diabetes management, where the emphasis is on the pattern and rate of glucose level changes rather than the exact numerical value, making the data more actionable for integrated algorithms^50,51^. Just as CGMS uses trends to dynamically adjust insulin delivery, MEC operation could benefit from similar strategies to determine varying threshold points and assess if the system is inhibited.
2. **Integration of Multi-Parameter Sensing:** Adding additional real-time sensors to the FSM can significantly enhance overall system performance and resilience. For instance, real-time methane monitoring provides immediate insights into AD performance. As shown in Figure 10 and Supplementary Figures S1–S2, under FSM control, methane content in the biogas remains stable at approximately 50–60% during continuous feeding, indicating consistent methanogenic activity. Each FSM-initiated feeding event corresponds to a spike in biogas flow, reflecting a microbial response. This is accompanied by sinusoidal changes in the MEC current draw. The close relationship between current and methane output underscores the value of monitoring both parameters, suggesting that integrating methane sensors into the FSM could further improve detection of digester states. Cross-referencing electrical and methane data helps the controller distinguish normal from abnormal conditions, thereby enhancing feeding decisions. It also paves the way for the development of self-recovery routines to automatically manage refeeding when process instability is detected.
  If sensor readings indicate an anomaly such as a feeding event that fails to produce the expected rise in current the FSM can transition the reactor into a protective recovery state. As illustrated in Figure 11, a spike in the FOS/TAC ratio (which signals excessive volatile acids relative to buffering capacity) or a sudden drop in methane concentration can be signs of system overload or inhibition.
  A robust recovery strategy can mitigate the adverse effects of prolonged feeding interruptions. Extended periods of nutrient deprivation can lead to cell death within the deeper layers of biofilm structures. This results in a residual layer composed of dead cells and extracellular substances, which effectively insulates and impedes electron transfer^52^. This can lead to a decrease in electroactive microbes and a drop in current output. Developing dedicated recovery state ensures feeding is reduced so that the digester stabilises, yet is brought back online without undue downtime. By integrating methane metrics and other sensor inputs directly into its state logic, the FSM effectively closes the loop on recovery, allowing the AD or MEC-AD system to stabilise after disturbances. During this phase, integrated methane sensing can confirm that methanogenesis is recovering for instance, by observing a steady rise back to 50–60% methane and the re-emergence of predictable methane production peaks after each feeding. Concurrently, electrode current should response, confirming biofilm reactivation. This sensor augmented, adaptive feeding control improves resilience against process failures and maintains optimal performance during normal operation by continuously aligning feeding decisions with the real-time metabolic status of the microbial community. Moreover, integrating sensors allows the FSM to autonomously detect failing states and automate recalibration strategies, including adjustments to current-based feeding thresholds.
3. **Adaptive Thresholds and Predictive Modelling:** Implementing machine learning algorithms within the FSM can allow for adaptive threshold settings that adjust in real-time based on the trends observed in response data. Further work may validate the effectiveness of integrating these predictions into the FSM to dynamically adjust thresholds based on emerging trends. This research will aim to quantify the impact on system stability, to reduce events where the determined threshold has not generated a stable system response. Further exploration will also assess the scalability of this method across different operational conditions such as varying OLR and HRT.
4. **Implementation of Learning for Adaptive Operation:** The enhanced FSM should continuously learn from operational data to improve its accuracy and reliability. By employing a feedback loop that records the success or failure of state transitions and adjusts its parameters accordingly. This continuous learning process could enable the system to autonomously manage operation periods such as the start-up phase by recognising patterns and making decisions using historical data and performance metrics, rather than relaying on a human data inspection to determine whether or not a feed cycle has been a successful. Consequently, operation periods that currently require regular sampling and lab analysis could be controlled more efficiently.
  **Validation Using Complex and Heterogeneous Feedstocks:** While molasses was selected as a controlled and readily biodegradable substrate to evaluate the FSM control framework, it represents a relatively simple carbohydrate source that rapidly hydrolyses to volatile fatty acids (VFAs). Under these conditions, hydrolysis is unlikely to be rate-limiting, and the relationship between soluble intermediates and anodic current response is comparatively direct. However, full-scale anaerobic digestion systems typically process heterogeneous substrates such as food waste, agricultural residues, and mixed organic slurries, which exhibit variable composition and slower hydrolysis kinetics. These factors may alter the temporal coupling between substrate degradation, VFA production, and electrochemical response.
  Future investigations should therefore evaluate the robustness of the biosensor-led control strategy using more complex and slowly hydrolysable feedstocks. Such studies would assess whether the current-based control thresholds remain reliable under conditions where hydrolysis dynamics, particulate content, and conductivity variability introduce additional system complexity. Controlled comparisons across substrates with differing biodegradability profiles would provide insight into how feedstock composition influences signal fidelity and feeding responsiveness.
  Validation under heterogeneous loading conditions will be essential for demonstrating scalability and practical deployment potential. Extending this framework to real waste streams would not only test system resilience but also strengthen the case for implementation in industrial anaerobic digestion facilities, where variability in feed composition is inherent. Establishing performance consistency across diverse substrates would significantly enhance confidence in the FSM-based control approach as a transferable and robust automation strategy.

Implementing these proposed modifications aims to enhance the resilience and operational efficiency of MEC-AD systems. Future updates to system design may support alignment with broader advancements in automation and machine learning and inform future investigation toward industrial implementation.

## Supplementary Information

Supplementary Information.

## Data Availability

Data will be made available on request. For requesting data, please write to the corresponding author.

## References

[1] Liew, P., Varbanov, P., Foley, A. & Klemeš,. Smart energy management and recovery towards sustainable energy system optimisation with bio-based renewable energy. *Renew. Sustain. Energy Rev.* **110385**, 110385. 10.1016/j.rser.2020.110385 (2021).

[2] Weiland,. Biogas production: current state and perspectives. *Appl. Microbiol. Biotechnol.* **7**, 849–860. 10.1007/s00253-009-2246-7 (2010). 19777226

[3] Wang, W., Chang, J.-S. & Lee,. Integrating anaerobic digestion with bioelectrochemical system for performance enhancement: A mini review. *Bioresource Technology* **126519**, 126519. 10.1016/j.biortech.2021.126519 (2022). 34896531

[4] Hassanein, A., Witarsa, F., Lansing, S., Qiu, L. & Liang,. Bio-electrochemical enhancement of hydrogen and methane production in a combined anaerobic digester (ad) and microbial electrolysis cell (mec) from dairy manure. *Sustainability* 8491. 10.3390/su12208491 (2020).

[5] Pawar, A., Karthic, A., Lee, S., Pandit, S. & Jung, S. Microbial electrolysis cells for electromethanogenesis: Materials, configurations and operations. *Environ. Eng. Res.* **27**, 10.4491/eer.2020.484 (2022).

[6] Zhao,. The underlying mechanism of enhanced methane production using microbial electrolysis cell assisted anaerobic digestion (mec-ad) of proteins. *Water Res.* **117325**, 117325. 10.1016/j.watres.2021.117325 (2021). 34144484

[7] Sleutels, T., Lodder, R., Hamelers, H. & Buisman,. Improved performance of porous bio-anodes in microbial electrolysis cells by enhancing mass and charge transport. *Int. J. Hydrogen Energy* **089**, 9655–9661. 10.1016/j.ijhydene.2009.09.089 (2009).

[8] Yu,. A review on the applications of microbial electrolysis cells in anaerobic digestion. *Bioresource Technology* **003**, 340–348. 10.1016/j.biortech.2018.02.003 (2018). 29444757

[9] Clauwaert, P. & Verstraete,. Methanogenesis in membraneless microbial electrolysis cells. *Appl. Microbiol. Biotechnol.* **4**, 829–836. 10.1007/s00253-008-1796-4 (2009). 19050859

[10] Radhika, D., Shivakumar, A., Kasai, D., Koutavarapu, R. & Peera,. Microbial electrolysis cell as a diverse technology: Overview of prospective applications, advancements, and challenges. *Energies* **15**, 2611. 10.3390/en15072611 (2022).

[11] Weithmann, et al. Flexible feeding in anaerobic digestion-impact on process stability, performance and microbial community structures. *Anaerobe* **102297**, 102297. 10.1016/j.anaerobe.2020.102297 (2021). 33212292

[12] Bonk, et al. Intermittent fasting for microbes: how discontinuous feeding increases functional stability in anaerobic digestion. *Biotechnol. Biofuels* **5**, 1–15. 10.1186/s13068-018-1279-5 (2018). PMC6173896 30323859

[13] Jegede, AeaAro et al. A review of mixing, design and loading conditions in household anaerobic digesters. *Crit. Rev. Environ. Sci. Technol.* **49**, 2117–2153. 10.1080/10643389.2019.1607441 (2019).

[14] Jimenez, et al. Instrumentation and control of anaerobic digestion processes: a review and some research challenges. *Reviews in Environmental Science and Bio/Technology* **648**, 615–648 (2015).

[15] Willeghems, G. & Buysse,. Changing old habits: The case of feeding patterns in anaerobic digesters. *Renew. Energy* **081**, 212–221. 10.1016/j.renene.2016.01.081 (2016).

[16] He, D., Wang, H., Tian, Y., Christov, N. & Simeonov,. Trajectory tracking of two-stage anaerobic digestion process: A predictive control with guaranteed performance and saturated input, based on ultra-local model. *J. Process Control* **103039**, 103039. 10.1016/j.jprocont.2023.103039 (2023).

[17] Gaida, D., Wolf, C. & Bongards, M. Feed control of anaerobic digestion processes for renewable energy production: A review. *Renew. Sustain. Energy Rev.* **68**, 869–875. 10.1016/j.rser.2016.06.096 (2017).

[18] Ahmed, W. & Rodríguez, J. A model predictive optimal control system for the practical automatic start-up of anaerobic digesters. *Water Res.* **174**, 115599. 10.1016/j.watres.2020.115599 (2020). 32086134

[19] Almansa, X. et al. Anaerobic digestion as a core technology in addressing the global sanitation crisis: challenges and opportunities. *Environ. Sci. & Technol.* **57**, 19078–19087. 10.1021/acs.est.3c05291 (2023). 37956995 PMC10702437

[20] Wang, Y., Huntington, T. & Scown, C. Tree-based automated machine learning to predict biogas production for anaerobic co-digestion of organic waste. *ACS Sustain. Chem. & Eng.* **9**, 12990–13000. 10.1021/acssuschemeng.1c04612 (2021).

[21] Zajkac, W., Andrzejewski, G., Krzywicki, K. & Królikowski, T. Finite state machine based modelling of discrete control algorithm in lad diagram language with use of new generation engineering software. *Procedia Comput. Sci.* **159**, 2560–2569. 10.1016/j.procs.2019.09.431 (2019).

[22] Jamhour, A. & García, C. Automation of industrial serial processes based on finite state machines. *Procedia Eng.* **42**, 186–196. 10.1016/j.proeng.2012.07.409 (2012).

[23] Amani, T., Nosrati, M. & Mousavi, S. M. Monitoring and process control in anaerobic digestion: A review. *Renew. Sustain. Energy Rev.* **14**, 2553–2561. 10.1016/j.rser.2010.07.072 (2010).

[24] Nguyen, D., Gadhamshetty, V., Nitayavardhana, S. & Khanal, S. Automatic process control in anaerobic digestion technology: A critical review. *Bioresour. Technol.* **193**, 513–522. 10.1016/j.biortech.2015.06.080 (2015). 26148991

[25] Poh, P., Gouwanda, D., Mohan, Y., Gopalai, A. & Tan, H. Optimization of wastewater anaerobic digestion using mechanistic and meta-heuristic methods: current limitations and future opportunities. *Water Conserv. Sci. Eng.* **1**, 1–20. 10.1007/s41101-016-0001-3 (2016).

[26] Gupta,. Review of explainable machine learning for anaerobic digestion. *Bioresource Technology* **128468**, 128468. 10.1016/j.biortech.2022.128468 (2023). 36503098

[27] Ling, J. et al. Machine learning methods for the modelling and optimisation of biogas production from anaerobic digestion: a review. *Environ. Sci. Pollut. Res.* **31**, 19085–19104. 10.1007/s11356-024-32435-6 (2024). 38376778

[28] Gladyshev, P. & Patel, A. Finite state machine approach to digital event reconstruction. *Digit. Investig.* 130–149, 10.1016/j.diin.2004.03.001 (2004).

[29] Baek, G., Saikaly, P. & Logan, B. Addition of a carbon fiber brush improves anaerobic digestion compared to external voltage application. *Water Res.* **188**, 116575. 10.1016/j.watres.2020.116575 (2021). 33152589

[30] Hussain, S., Perrier, M. & Tartakovsky, B. Long-term performance of a microbial electrolysis cell operated with periodic disconnection of power supply. *RSC Adv.* **8**, 16842–16849. 10.1039/C8RA01863D (2018). 35540527 PMC9080321

[31] Flores-Rodriguez, C., Reddy, C. & Min, B. Enhanced methane production from acetate intermediate by bioelectrochemical anaerobic digestion at optimal applied voltages. *Biomass and Bioenergy* 105261, 10.1016/j.biombioe.2019.105261 (2019).

[32] Choi, K.-S., Kondaveeti, S. & Min, B. Bioelectrochemical methane () production in anaerobic digestion at different supplemental voltages. *Bioresour. Technol.* 826–832, 10.1016/j.biortech.2017.09.057 (2017). 28926915

[33] Banu, J., Kaliappan, S. & Beck, D. Treatment of molasses wastewater in an anaerobic reactor. *Process. Biochem.* **41**, 707–713 (2006).

[34] Akca, M. S., Ceylan-Perver, G., Iren, E. & Altinbas, M. Anaerobic co-digestion of landfill leachate as main energy source. *Int. J. Environ. Sci. Technol.* **21**, 6871–6890. 10.1007/s13762-023-05441-3 (2024).

[35] Lipps, W., Braun-Howland, E. & Baxter, T. *Standard Methods for the Examination of Water and Wastewater* (American Public Health Association, 2023).

[36] Martí-Herrero, J., Flores, T., Alvarez, R. & Perez, D. How to report biogas production when monitoring small-scale digesters in field. *Biomass and Bioenergy* 31–36, 10.1016/j.biombioe.2015.11.004 (2016).

[37] Jukuri, S., Bastipati, S., Dheravath, B. & Lavudi, S. Biochemical process evaluation of an anaerobic digester: a case study on long sustain commercial biogas plant. *Biomass Convers. Biorefinery* 1–10, 10.1007/s13399-021-01410-3 (2022).

[38] Dave, S. et al. Contribution of cyclic voltammetry and electrochemical impedance spectroscopy in deciphering the electron transport system in biofilm. In *Analytical Methodologies for Biofilm Research*, 129–152, 10.1007/978-1-0716-1378-8_6 (Springer, 2021).

[39] Dubrovin, I. et al. Hydrogen production in microbial electrolysis cells based on bacterial anodes encapsulated in a small bioreactor platform. *Microorganisms* **10**, 1007. 10.3390/microorganisms10051007 (2022). 35630450 PMC9142973

[40] Xu, S., Zhang, Y., Luo, L. & Liu,. Startup performance of microbial electrolysis cell assisted anaerobic digester (mec-ad) with pre-acclimated activated carbon. *Bioresour. Technol. Rep.* **007**, 91–98. 10.1016/j.biteb.2018.12.007 (2019). PMC6524652 31193294

[41] Ghaderikia, A. Start-Up Strategies for Enhanced Methane Production from Cattle Manure in Bioelectrochemical Systems (Middle East Technical University (Turkey, 2022). 10.1016/j.wasman.2023.01.027 (Master&apos;s thesis).

[42] Tran, H. V., Kim, E. & Jung, SPAbm. Anode biofilm maturation time, stable cell performance time, and time-course electrochemistry in a single-chamber microbial fuel cell with a brush-anode. *J. Ind. Eng. Chem.* **001**, 269–278. 10.1016/j.jiec.2021.11.001 (2022).

[43] Sapireddy, V., Katuri, K. P., Muhammad, A. & Saikaly, P. E. Competition of two highly specialized and efficient acetoclastic electroactive bacteria for acetate in biofilm anode of microbial electrolysis cell. *npj Biofilms and Microbiomes* **7**, 47, 10.1038/s41522-021-00218-3 (2021). PMC8166840 34059681

[44] Litti, et al. Electromethanogenesis: a promising biotechnology for the anaerobic treatment of organic waste. *Appl. Biochem. Microbiol.* **58**, 19–36. 10.1134/S0003683822010057 (2022).

[45] Usman, M., Salama, E.-S., Arif, M., Jeon, B.-H. & Li,. Determination of the inhibitory concentration level of fat, oil, and grease (fog) towards bacterial and archaeal communities in anaerobic digestion. *Renew. Sustain. Energy Rev.* **110032**, 110032. 10.1016/j.rser.2020.110032 (2020).

[46] Yuan, H. & Zhu,. Progress in inhibition mechanisms and process control of intermediates and by-products in sewage sludge anaerobic digestion. *Renew. Sustain. Energy Rev.* **261**, 429–438. 10.1016/j.rser.2015.12.261 (2016).

[47] Xu, et al. The effect of pbs on methane production in combined mec-ad system fed with alkaline pretreated sewage sludge. *Renew. Energy* **052**, 229–236. 10.1016/j.renene.2020.01.052 (2020).

[48] Li, Y., Chen, Y. & Wu,. Enhancement of methane production in anaerobic digestion process: A review. *Appl. Energy* **243**, 120–137. 10.1016/j.apenergy.2019.01.243 (2019).

[49] Zakaria, B. S., Lin, L., Chung, T. & Dhar,. An overview of complementary microbial electrochemical technologies for advancing anaerobic digestion. *Adv. Bioenergy* **004**, 129–167. 10.1016/bs.aibe.2020.04.004 (2020).

[50] Rodbard, DCgmaros. Continuous glucose monitoring: a review of successes, challenges, and opportunities. *Diabetes Technol. Ther.* **0417**, S2-3. 10.1089/dia.2015.0417 (2016). PMC4717493 26784127

[51] Heise, T., Koschinsky, T., Heinemann, L. & Lodwig,. Hypoglycemia warning signal and glucose sensors: requirements and concepts. *Diabetes Technol. Ther.* **5**, 563–571. 10.1089/152091503322250587 (2003). 14511411

[52] Jha, S. & Anand,. Development and control of biofilms: novel strategies using natural antimicrobials. *Membranes* **13**, 579. 10.3390/membranes13060579 (2023). PMC10302042 37367783

