# Supplementary material for: Automated real-time feeding control for microbial electrolysis cell-anaerobic digestion systems using finite state machine
Source: Sci Rep. 2026 Jun 20;16:28260. doi: 10.1038/s41598-026-57116-x (PMC13558775; doi:10.1038/s41598-026-57116-x)
Supplement: Supplementary file 1 [file 41598_2026_57116_MOESM1_ESM.pdf]

Supplementary Figures

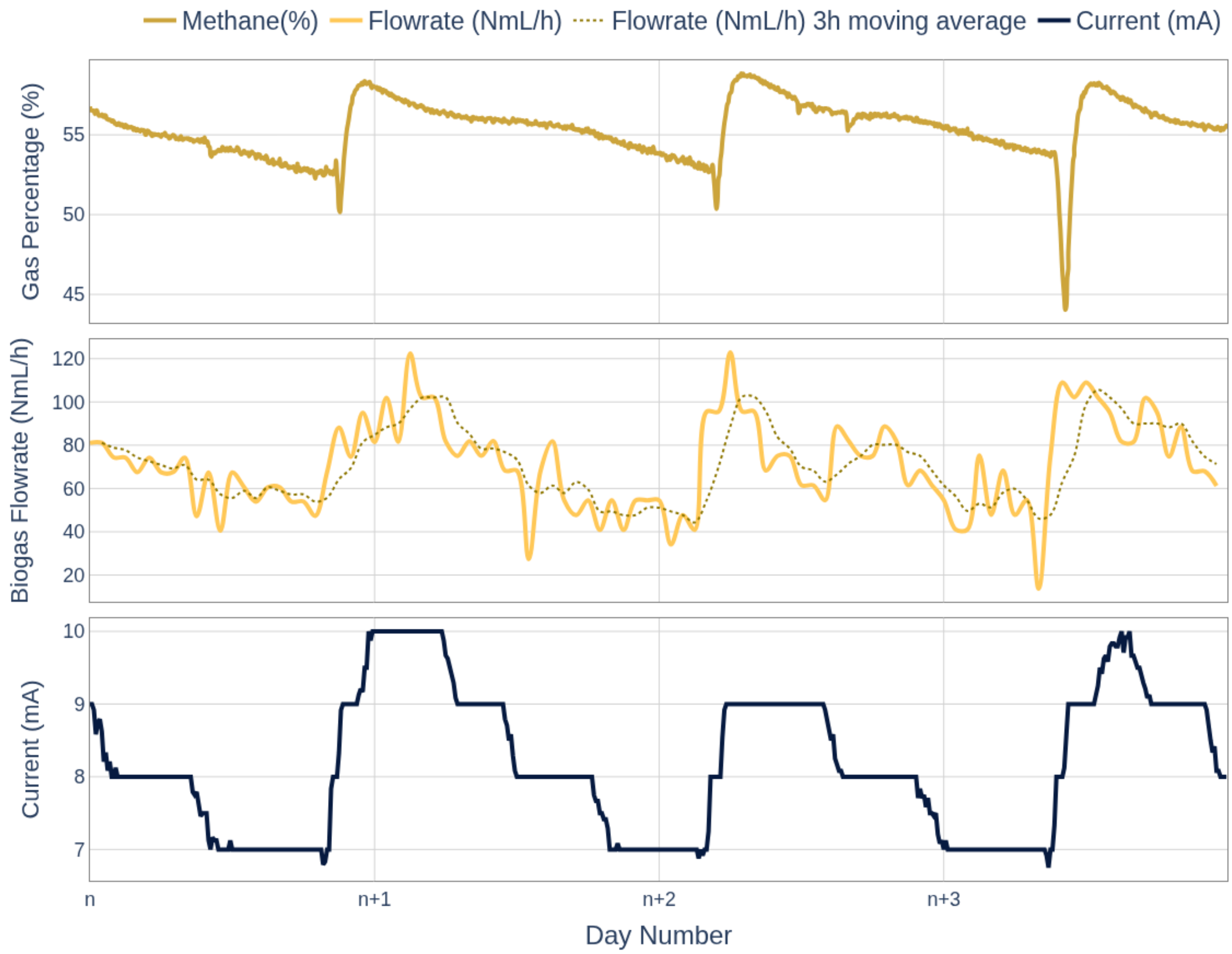

Figure 1. Figure S1. Time-series response of Reactor 1 showing current draw, biogas production, and methane composition during FSM-controlled operation.

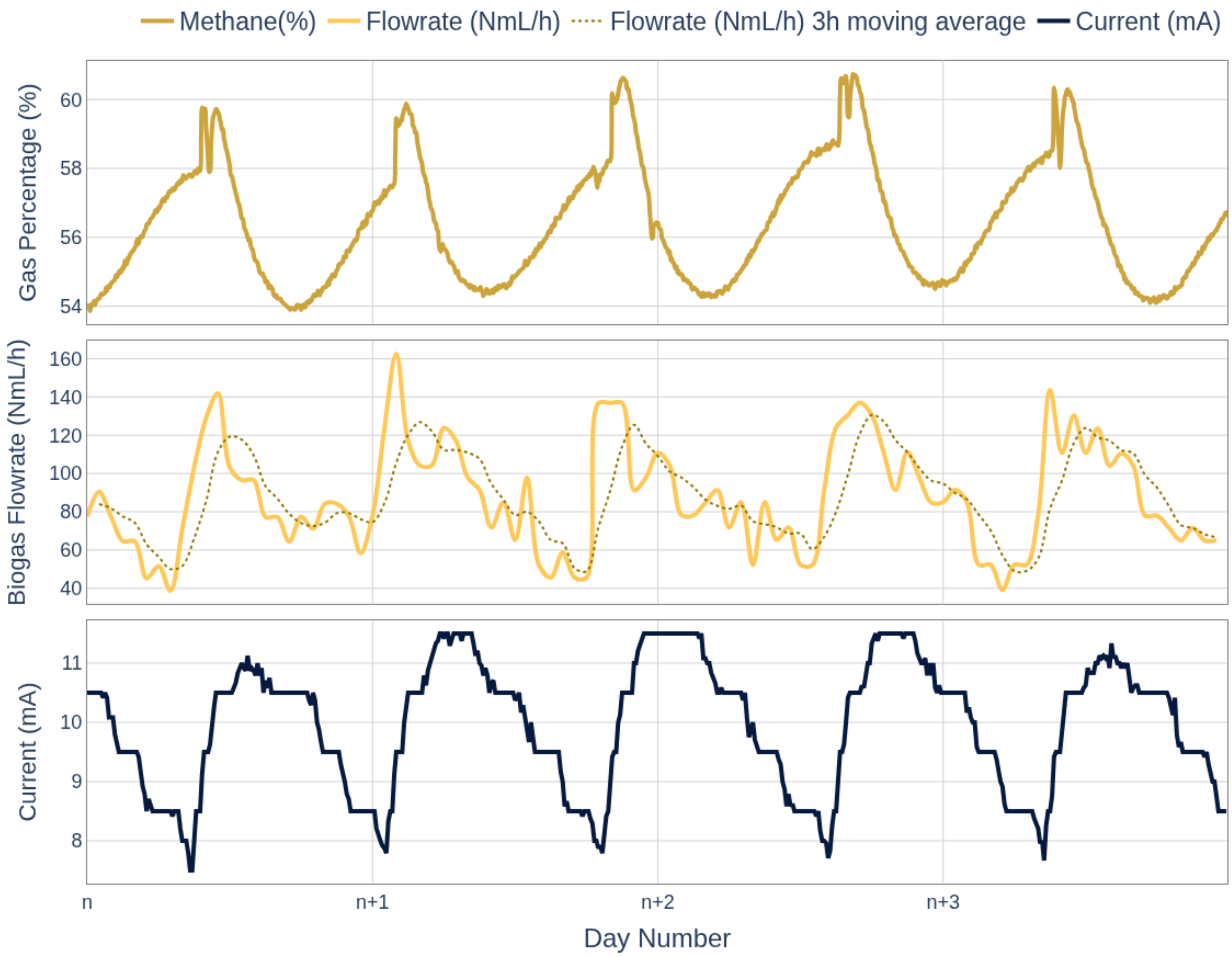

Figure 2. **Figure S2.** Time-series response of Reactor 2 showing current draw, biogas production, and methane composition during FSM-controlled operation.
